# Multi-strategy remora optimization algorithm for color multi-threshold image segmentation

**DOI:** 10.1371/journal.pone.0342261

**Published:** 2026-02-18

**Authors:** Heming Jia, Changsheng Wen, Honghua Rao, Laith Abualigah, Mahmoud Abdel-Salam

**Affiliations:** 1 School of Information Engineering, Sanming University, Sanming, Fujian, China; 2 College of Information and Electrical Engineering, Heilongjiang Bayi Agricultural University, Daqing, Heilongjiang, China; 3 School of Electrical and Information Engineering, Northeast Petroleum University, Daqing, Heilongjiang, China; 4 Hourani Center for Applied Scientific Research, Al-Ahliyya Amman University, Amman, Jordan; 5 Faculty of Computers and Information Science, Mansoura University, Mansoura, Egypt; SR University, INDIA

## Abstract

Image segmentation is a fundamental step in image processing, yet determining the optimal thresholds for multi-threshold segmentation remains a computationally challenging task as the search space expands exponentially with the number of thresholds. To effectively address this issue, this paper proposes a Multi-Strategy Remora Optimization Algorithm (MSROA) designed for efficient color image segmentation. MSROA improves upon the standard algorithm by integrating a Beta random restart strategy with a “prior” property to prevent stagnation in local optima, alongside a random walk with fast predation and an elite learning strategy to enhance convergence speed and solution accuracy. The optimization performance of MSROA was rigorously evaluated on the CEC2017 and CEC2020 benchmark test suites. Wilcoxon rank-sum tests confirmed that MSROA achieves statistically significant improvements over seven state-of-the-art comparison algorithms. Furthermore, the algorithm was applied to color image segmentation tasks using Otsu’s method and Kapur’s entropy as objective functions. Experimental results on standard datasets demonstrate that MSROA not only identifies optimal threshold combinations more accurately but also yields segmented images with superior quality. Quantitative evaluations show that MSROA consistently achieves higher Peak Signal-to-Noise Ratio (PSNR), Feature Similarity Index Measure (FSIM), and Structural Similarity Index Measure (SSIM) values compared to competitors, proving its capability to effectively preserve fine textures and edge details even at high threshold levels. The source code of MSROA is publicly available at https://github.com/wencs666/MSROA.

## 1 Introduction

Image segmentation [1] is a fundamental technique in computer vision and image processing, defined as the process of partitioning an image into distinct regions where pixels share high similarity in attributes such as color, intensity, or texture. The importance of this process cannot be overstated, as it serves as a prerequisite step that directly influences the performance of downstream analysis tasks. For instance, in the medical field, precise segmentation is crucial for lesion detection and identifying diseased tissues, thereby assisting doctors in accurate diagnosis [2]. In intelligent transportation systems, it enables vehicle detection [3] and traffic congestion analysis [4], reducing the burden on traffic management. Similarly, in precision agriculture [5,6] and industrial quality control [7,8], the efficiency of production and monitoring relies heavily on the accuracy of automated segmentation. Consequently, developing robust segmentation methods is a critical research priority with wide-ranging applications in remote sensing [9], facial recognition [10], and aerospace technology [11].

Image segmentation can be categorized into color image segmentation [12] and grayscale image segmentation [13] based on the type of input image. According to segmentation criteria, it can be further classified into threshold-based methods [14], region-based methods [15], and edge-based methods [16]. In addition, there are other methods developed based on specific theories [17]. Among these, threshold-based segmentation techniques are commonly divided into two types: single-threshold segmentation and multi-threshold segmentation. Single-threshold segmentation classifies pixel grayscale values into two groups—foreground (target region) and background—based on a single threshold value. In contrast, multi-threshold segmentation uses multiple thresholds to divide the image into several distinct regions. Compared to single-threshold segmentation, multi-threshold segmentation is more effective in handling complex backgrounds and images with multiple target regions, making it widely applicable in various image analysis tasks.

Numerous methods have been developed to address image segmentation problems. For instance, the Kapur entropy method [18], also known as the maximum entropy method, segments the image histogram into multiple regions using thresholds and seeks to maximize the sum of the entropies of these regions. The Otsu method [19], or maximum between-class variance method, selects thresholds by maximizing the variance between segmented classes. In addition to these, several techniques have been proposed for multi-threshold image segmentation, including the minimum cross-entropy method (MCE) [20]. However, as the number of thresholds increases, the search space expands rapidly, making it increasingly difficult to identify optimal thresholds using traditional exhaustive search methods. To overcome this challenge, swarm intelligence-based optimization algorithms have gained significant attention in recent years for effectively solving multi-level threshold image segmentation problems.

Swarm intelligence-based optimization algorithms, inspired by various natural and social phenomena, can be broadly categorized into four groups based on their underlying inspiration: physics-based, evolutionary-based, swarm-based, and human-based algorithms. Physics-based meta-heuristic algorithms simulate physical laws and principles to guide the search process. Representative examples include the Big-Bang Big Crunch (BB-BC) algorithm [21], Lightning Search Algorithm (LSA) [22], Artificial Electric Field Algorithm (AEFA) [23], Sine Cosine Algorithm (SCA) [24], Arithmetic Optimization Algorithm (AOA) [25], Gravitational Search Algorithm (GSA) [26], Black Hole Algorithm (BHA) [27], and Henry Gas Solubility Optimization (HGSO) [28]. Evolutionary-based algorithms are inspired by biological evolution mechanisms, such as the Artificial Algae Algorithm (AAA) [29], Genetic Algorithm (GA) [30], Monkey King Evolution (MKE) [31], and Differential Evolution (DE) [32]. Swarm-based algorithms model the collective behavior of social organisms, including Particle Swarm Optimization (PSO) [33], Moth-Flame Optimization (MFO) [34], Ant Colony Optimization (ACO) [35], Whale Optimization Algorithm (WOA) [36], and Marine Predator Algorithm (MPA) [37]. Human-based algorithms mimic human social behavior and decision-making strategies, such as the Imperialist Competitive Algorithm (ICA) [38], Cohort Intelligence (CI) [39], and Social Group Optimization (SGO) [40]. According to the No Free Lunch (NFL) theorem [41], no single algorithm performs best across all optimization problems. This has driven the continuous development of novel algorithms and improvements to existing ones. Consequently, numerous enhanced or hybrid variants have been proposed in recent years, including MGTO [42], IWHO [43], MHHO [44], and MSCSO [45].

At present, many researchers have applied swarm intelligence optimization algorithms to multi-threshold image segmentation problems. Rao et al. enhanced the performance of the crayfish optimization algorithm by optimizing the maximum foraging amount parameter, introducing an adaptive foraging adjustment strategy, and incorporating the core formula of the differential evolution algorithm [46]. Their method demonstrated excellent performance in multi-threshold image segmentation tasks. Jia et al. improved the artificial rabbit optimization algorithm by integrating a center-driven strategy and a Gaussian random walk mechanism to enhance its optimization capability. When applied to multi-threshold color image segmentation, their approach achieved high segmentation accuracy and fast execution [47]. Peng et al. proposed a hybrid algorithm by improving the dragonfly optimization algorithm through chaotic initialization and elite reverse learning, and integrating it with the differential evolution algorithm. They employed Kapur entropy, Minimum Cross-Entropy, and Otsu methods as objective functions to obtain optimal fitness values. The segmented images achieved high performance in terms of pixel similarity, structural similarity, brightness, and contrast [48]. Ma et al. optimized the initial population of the whale optimization algorithm using reverse learning, introduced an adaptive factor to balance exploration and exploitation, and incorporated horizontal and vertical crossover strategies to enhance optimization performance [49]. Their method achieved the highest similarity between segmented and original images, delivering higher-quality solutions and greater stability.

The Remora Optimization Algorithm (ROA) [50] is a meta-heuristic swarm intelligence algorithm inspired by the foraging behavior of remoras. The core idea behind ROA is to adaptively select different host-switching and behavioral strategies based on various stages of the remora’s foraging process. However, standard ROA faces specific limitations when applied to the complex multimodal landscapes of multi-threshold segmentation. It is prone to premature convergence, often getting trapped in local optima during the early search phase, and suffers from slow convergence rates. Although improved versions exist [51–53], challenges related to insufficient global exploration and the randomness of roulette wheel selection remain, potentially causing the algorithm to miss global optima.

However, despite these improvements, existing variants of ROA still face challenges related to exploration and convergence. For example, the MROA does not sufficiently address exploration, leading to slower convergence rates. Additionally, the randomness inherent in roulette wheel selection may cause the algorithm to skip over optimal solutions during the search process. To overcome these shortcomings, this paper introduces a Multi-Strategy Remora Optimization Algorithm (MSROA). First, a Beta Random Restart Strategy is proposed, which leverages the “prior” properties of the beta distribution to simulate evolutionary exploration and elimination, helping the algorithm escape local optima. Second, a two-phase strategy is introduced, combining random walk in the early stage (simulating autonomous host exploration) with fast predation in the later stage (simulating rapid target capture). This approach not only increases population diversity but also improves convergence accuracy. Finally, inspired by different learning behaviors, an elite learning strategy is developed, combining elite forward learning and elite reverse learning to effectively mitigate the negative impact of local optima.

Through the integration of these strategies, MSROA achieves both enhanced global exploration and faster convergence. The main contributions of this paper are as follows:

- A Beta Random Restart Strategy is proposed by improving the traditional restart mechanism. Inspired by biological exploration and leveraging the ‘prior’ properties of the beta distribution, this strategy effectively enhances the algorithm’s ability to escape local optima and significantly improves convergence speed.
- A Two-Phase Search Mechanism is introduced by simulating different predation behaviors of the host. The random walk in the early stage enhances exploration, while the fast predation in the later stage strengthens exploitation, thereby improving the overall optimization capability of the algorithm.
- An Elite Learning Strategy is designed by mimicking two distinct learning behaviors—elite forward learning and elite reverse learning. This strategy mitigates the negative effects of premature convergence to local optima and improves the accuracy and robustness of the final solutions.

To evaluate the optimization performance of the proposed MSROA, this study conducted experiments using 29 benchmark functions from the CEC 2017 test suite and 10 benchmark functions from the CEC 2020 test suite. Finally, MSROA was applied to solve multi-threshold image segmentation problems. The segmented images obtained using MSROA not only achieved superior fitness values but also outperformed competing methods in terms of Peak Signal-to-Noise Ratio (PSNR), Feature Similarity Index Measure (FSIM), and Structural Similarity Index Measure (SSIM), demonstrating improved segmentation quality across multiple evaluation metrics.

The rest of this paper is organized as follows: Sect 2 describes the original algorithm of ROA. Sect 3 introduces the Beta random restart strategy, random walk, fast predation, and elite learning strategy. Sect 4 introduces the theory and methods of the multi-threshold image segmentation problem. Sect 5 presents the experimental results and analysis of MSROA on CEC benchmark functions and its application effects in image segmentation tasks. Sect 6 summarizes this paper and discusses future research directions.

## 2 Remora optimization algorithm (ROA)

The Remora Optimization Algorithm (ROA) is a novel swarm intelligence optimization algorithm introduced by Jia et al. in recent years, inspired by the foraging behavior of remoras. Initially, the remora selects a host and attaches itself to it, obtaining the necessary food through this attachment. The remora then switches between different hosts, such as swordfish and whales, based on its experience. To further enhance its food acquisition, the remora engages in host foraging, attacking different hosts to optimize its resource collection. In this process, the SFO (Swordfish Foraging Optimization) algorithm is employed during the exploration phase, while the WOA (Whale Optimization Algorithm) is utilized in the exploitation phase.

### 2.1 Initialization

In the initialization phase, a population of random candidate solutions is generated within the defined search space, where each individual serves as an initial solution for the ROA. The initialization process is mathematically described by Eq (1) as follows:

$$X_{i}=lb+\text{rand}\times (ub-lb)$$ (1)

where, *X*_*i*_ is the position of the solution, *lb* and *ub* are the lower and upper bounds of the search space, respectively, and *rand* is a random number that can be evenly distributed between 0 and 1.

### 2.2 Free travel (Exploration)

#### 2.2.1 SFO strategy.

During the exploration phase, the SFO algorithm updates the position of the swordfish. Since the remora is in the exploration stage, its movement speed is faster. The remora selects the swordfish as its host, attaches to it, and follows its movement. The position update in this stage is governed by Eq (2):

$$X_{i}^{t+1}=X_{Best}^{t}-(\text{rand}\times (\frac{X_{Best}^{t}+X_{rand}^{t}}{2})-X_{rand}^{t})$$ (2)

where *t* represents the current iteration number, $X_{\text{Best}}^{t}$ is the position of the current optimal individual, and $X_{\text{rand}}^{t}$ is the position of a randomly selected fish from the population.

#### 2.2.2 Experience attack strategy.

When the remora is in the exploration stage, a small range of exploratory attacks will be carried out according to the host’s location and the previous generation of remoras. The updated formula of remora is shown in Formula (3):

$$X_{att}={X_{i}}^{t}+\text{(}{{\text{X}}_{i}}^{t}+X_{pre}\text{)}\times \text{randn}$$ (3)

where $X_{\text{att}}$ is the exploratory motion attack of the remora, $X_{\text{pre}}$ is the position of the previous generation of the remora, and randn is a random number that follows a normal distribution between 0 and 1.

After a small number of attempts, Formula (4) determines whether the host needs to be switched. The formula for switching the host is shown in Formula (5):

$$f(X_{i}^{t})<f(X_{att})$$ (4)

H(i)=round(rand) (5)

where, the remora is selected to adsorb on the host by *H*(*i*), and the initial value is 0 or 1. If *H*(*i*) equals 0, the remora adsorb on the whale. If *H*(*i*) equals 1, the remora adsorb on the swordfish. The round represents a rounded function, $f(X_{i}^{t})$ represents the fitness value of $X_{i}^{t}$, and *f*(*X*_*att*_) represents the fitness value of *X*_*att*_.

### 2.3 Eat thoughtfully (Exploitation)

#### 2.3.1 WOA strategy.

When the remora is in the development stage, it adsorbs onto the whale and moves with it. According to the WOA, the position update of the remora is computed using the following Formulas (6, 7, 8, 9):

$${X_{i}}^{t+1}=D\times e^{k}\times \cos\text{(}2\pi a\text{)}+{X_{i}}^{t}$$ (6)

$$D=\left|{X_{Best}}^{t}-{X_{i}}^{t}\right|$$ (7)

k=rand×(a−1)+1 (8)

$$a=-\text{(}1+\frac{t}{T}\text{)}$$ (9)

where *D* is the distance between the position of the best individual and the position of the current individual, k∈[−1,1] is a random number, *a* is a control parameter that linearly decreases from –2 to 1 over the course of iterations, and *T* is the maximum number of iterations.

#### 2.3.2 Host feeding.

When the remora is in the development stage, it will perform a small-range local search near the host. The position update during this stage is described by the following formulas:

$${X_{i}}^{t+1}={X_{i}}^{t}+A$$ (10)

$$A=B\times \text{(}X_{i}^{t}-C\times X_{Best}\text{)}$$ (11)

B=2×V×rand−V (12)

$$V=2\times \text{(}1-\frac{t}{T}\text{)}$$ (13)

where *A* represents the distance between the previous position and the current position of the fish. To constrain the position updates, a constant factor *C* = 0.1 is used. To simulate the size of the host and the remora, *B* and *V* are introduced to represent the volume of the host and the volume of the remora, respectively.

**Fig 1:**
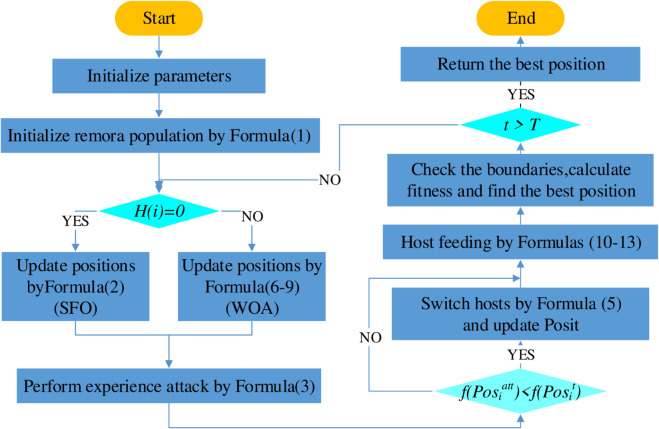
ROA flow chart.

The flowchart of ROA is presented in Fig 1.

## 3 Proposed method

### 3.1 Beta random restart strategy

Traditional restart strategies typically perform restarts after a fixed number of iterations. However, such strategies cannot promptly eliminate poor-performing individuals, and may also interrupt promising search trajectories of well-performing individuals. Additionally, an accumulation of poor individuals can lead the algorithm to become trapped in local optima.

To address these issues, this paper introduces a Beta random restart strategy inspired by biological evolutionary exploration. This strategy incorporates a *prior* property: when the number of iterations exceeds a threshold $T_{\text{beta}}$, the population is sorted based on fitness values, and the bottom half of individuals (i.e., poor solutions) are re-initialized. This approach ensures that unproductive individuals are refreshed, while preserving high-quality individuals to continue exploitation, thereby improving both convergence accuracy and speed.

The Beta distribution is selected over other common distributions, such as Gaussian or Cauchy, due to its specific mathematical properties and biological relevance. Mathematically, the Beta distribution is naturally defined on the bounded interval [0,1]. This aligns perfectly with the normalized timeline of the optimization process (represented by the ratio of current to maximum iterations), whereas Gaussian or Cauchy distributions are defined on an infinite domain (−∞,+∞), which would require artificial truncation or mapping to fit the search parameters.

Biologically, the Beta distribution serves as a “prior” probability model that simulates the uncertainty of the remora’s decision-making. Controlled by the random parameters *α* and *β*, the Beta distribution exhibits high shape flexibility—it can be symmetric, skewed left, or skewed right. This flexibility allows *T*_*beta*_ to dynamically vary, simulating the unpredictable environmental pressures that cause a remora to abandon a host at different stages of foraging. Unlike a rigid Gaussian bell curve, the Beta distribution allows the algorithm to explore various restart timings effectively.

The mathematical formulation of the Beta random restart strategy is shown as follows:

$$T_{beta}=Beta\left(\alpha ,\beta \right)\times T$$ (14)

α=5×rand (15)

β=5×rand (16)

$${X_{i}}^{\text{inferior}}=lb+\left(ub-lb\right)\times rand$$ (17)

where, $T_{\text{beta}}$ represents the number of random iterations, $X_{\text{i}}^{inferior}$ represents the position of the *i*-th “not good” individual, Beta(α,β) is a random number subject to Beta(α,β), and rand is a random number subject to a uniform distribution in (0,1). Note that *lb* and *ub* represent the lower and upper bounds of the search space, respectively.

### 3.2 Random walk with Fast predation strategy

ROA accelerates the convergence speed of the algorithm when simulating the experience attack and host switching of the remora. Still, it also makes the algorithm easy to fall into the local optimal solution, affecting the algorithm’s convergence accuracy. In order to enhance the exploration ability of the algorithm and improve the convergence speed of the algorithm, this paper simulates the purposeless search of the host in the first half stage. It simulates the fast predation behavior of the host in the second half stage.

At the same time, in the fast predation stage, this paper designs a nonlinear driving factor, which effectively accelerates the convergence speed of the algorithm. The factor *f* exhibits an increasing non-linear trend, serving to dynamically adjust the search step size. Specifically, in the early phase of the search (when *t* is small), *f* takes a value close to 0, resulting in smaller step sizes that facilitate stable, fine-grained exploration. As the iteration *t* progresses towards the maximum *T*, *f* gradually increases to 1. During the fast predation phase (where *t* > 0.5*T*), *f* ranges from approximately 0.37 to 1. This increasing magnitude allows the remora to make larger, more aggressive movements towards the global optimal solution in the final stages, thereby ensuring rapid convergence. The mathematical formula of random walk and fast predation strategy is as follows:

$$X_{new}=\{\begin{array}{cc} X_{{\;\;}_{best}}^{t}+rand\times \left(X_{{\;\;}_{rand}}^{1}-X_{{\;\;}_{rand}}^{2}\right) & t⩽0.5T\\ X_{i}^{t}+f\times X_{best}\times randi\left(\left[\begin{array}{cc} -1 & 1 \end{array}\right]\right) & t>0.5T \end{array}$$ (18)

$$f=\exp\left(1-\frac{T}{t}\right)$$ (19)

where, $X_{\text{new}}$ represents the position obtained by random walk and fast predation strategy; $X_{\text{rand}}^{1}$ and $X_{\text{rand}}^{2}$ represent the positions of two random individuals, *f* is the nonlinear driving factor of fast predation stage.

### 3.3 Elite learning strategy

Learning behavior can be the advantage of learning elites, but it also can be found its shortcomings, to improve themselves. Based on the inspiration of this learning behavior, simulating the advantages and disadvantages of learning elites, this paper proposes an elite learning strategy to strengthen the learning ability of remora. At the same time, elites must have many advantages because they are excellent. Therefore, we add learning factors and inhibitory factors to learning strategies. The learning factor fluctuates with the number of iterations in the early stage. With the deepening of learning, the later learning factor will gradually increase and strengthen the learning of elites. The inhibition factor decreases monotonously with the number of iterations, indicating that it reduces the impact of its shortcomings in the learning process. In the algorithm, the learning factor can accelerate the convergence speed of the algorithm, and the suppression factor can reduce the negative impact of the local optimal solution. The mathematical formula of the elite learning strategy is as follows:

$$X_{positive}=b\times X_{i}^{t}+sf\times X_{best}^{t}$$ (20)

$$X_{negative}=b\times X_{i}^{t}-sf\times X_{best}^{t}$$ (21)

$$b=\ln\left(1-\frac{t}{T}+rand\right)$$ (22)

$$sf=\exp\left[\sin\left(1-\frac{T}{t}+rand\right)\right]$$ (23)

where, $X_{\text{positive}}$ and $X_{\text{negative}}$ represent the position of forward learning and reverse learning, respectively. *sf* is the learning factor.

### 3.4 The proposed MSROA

**Fig 2:**
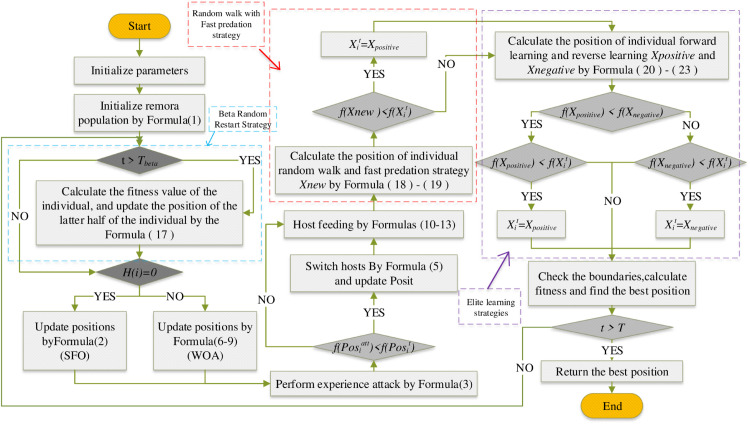
MSROA flow chart.

To further enhance the optimization performance of the ROA, this paper introduces a multi-strategy Remora Optimization Algorithm (MSROA). First, by simulating exploration in biological evolution, a Beta random restart strategy is proposed to improve the exploration capability of the algorithm and mitigate the impact of local optima. Next, through a combination of random walk and fast predation strategies, the movement and predation behavior of the host are further simulated, leading to an overall enhancement of the algorithm’s optimization performance. Finally, inspired by different learning behaviors, an elite learning strategy is introduced to improve convergence accuracy and accelerate the algorithm’s convergence speed. The pseudocode for the proposed MSROA is provided in Algorithm 1, and the corresponding flowchart is presented in Fig 2.

**Algorithm 1 MSROA algorithm pseudo code.**

1: Initialization parameters(Population size: *n*, maximum number of iterations: *T*, remora factor: *C*)

2: Initialize the population by Formula (1)

3: **while** *t* < *T* **do**

4:   Pulls individuals beyond the search space back to the boundary

5:   Calculate the fitness value of each individual and update *X*_*best*_

6:   **if** *t* > *T*_*rand*_ **then**

7:    The position of the second half of the individual is updated by Formula (17).

8:   **end if**

9:   **for** *i* = 1 to *n* **do**

10:    **if** H(i)==0 **then**

11:     Using Formula (2) to update the position of the individual.

12:    **else if** H(i)==1 **then**

13:     Using Formula (6) to update the position of the individual.

14:    **end if**

15:    Empirical attack according to Formula (3)

16:    **if** $f(X_{an})<f(X_{i})$ **then**

17:     Switch the host through Formula (5) and update *X*_*i*_

18:    **else**

19:     Host feeding by Formulas (10)

20:    **end if**

21:    Calculate the position of *X*_*new*_ by Formula (18).

22:    **if** $f(X_{new})<f(X_{i})$ **then**

23:     $X_{i}=X_{new}$

24:    **end if**

25:    Calculate the position of $X_{positive}$ and $X_{negative}$ by Formula (20) and (21).

26:    **if** $f(X_{positive})<f(X_{negative})$ **then**

27:     **if** $f(X_{positive})<f(X_{i})$ **then**

28:      $X_{i}=X_{positive}$

29:     **end if**

30:    **else if** $f(X_{negative})<f(X_{i})$ **then**

31:     $X_{i}=X_{negative}$

32:    **end if**

33:   **end for**

34:   *t* = *t* + 1

35: **end while**

36: **return** *X*_*best*_

### 3.5 Analysing algorithm complexity

The complexity of MSROA primarily arises from the initialization process, location updates, and fitness evaluations. The complexity of the initialization is O(N × dim), where *N* represents the population size and dim is the dimensionality of the search space. The complexity of location updates is O(N×dim×MaxFEs), where MaxFEs refers to the maximum number of fitness evaluations. The complexity of fitness evaluation is O(N×C×MaxFEs), where *C* is the time cost of evaluating a single solution.

Regarding the proposed learning strategy, the algorithm evaluates both positive and negative learning positions simultaneously. While this mechanism effectively doubles the number of function evaluations for the individuals involved in this specific phase within a single iteration, it remains within an acceptable computational range. In the asymptotic complexity analysis, the constant factor introduced by these additional evaluations (e.g., 2×N) does not alter the order of magnitude. Furthermore, the total computational cost is bounded by the maximum number of function evaluations (MaxFEs). Therefore, despite the increased evaluations per iteration, the overall algorithmic complexity class remains unchanged. Thus, the total time complexity of MSROA is:


O(N×dim+N×MaxFEs×(dim+C))

Comparing this with the traditional ROA, the time complexity has not increased, indicating that the additional strategies in MSROA do not significantly impact the overall computational cost.

## 4 Image segmentation theory and methods

Image segmentation based on thresholding aims to determine the optimal threshold using a specific method, and then compare the image pixels to distinguish the target from the background. Threshold-based image segmentation methods can be categorized into two main types: single-threshold segmentation and multi-threshold segmentation. In single-threshold segmentation, the image histogram is divided into two categories—target and background—based on a single threshold. In contrast, multi-threshold segmentation divides the image into multiple categories, aiming to maximize the inter-class variance between them. When the image is complex and contains multiple objects, the performance of the single-threshold method tends to be insufficient, which is why multi-threshold image segmentation has been widely explored.

In the context of color image segmentation addressed in this paper, it is crucial to clarify the processing strategy for color channels. Unlike methods that convert color images into a grayscale version, which may lead to the loss of potential color information, this study calculates the thresholds for the Red (R), Green (G), and Blue (B) channels individually. Specifically, the histogram for each RGB component is analyzed separately, and the optimal thresholds are determined by maximizing the objective function (Otsu or Kapur) for each channel independently. This component-wise approach ensures that the segmentation process fully leverages the color distribution details of the original image.

Several methods exist for multi-threshold image segmentation. In the following section, two such methods—the Otsu method and the Kapur entropy method—are discussed.

### 4.1 Otsu method (maximum between-class variance method)

The Otsu method was proposed by scholar Nobuyuki Otsu in 1979 [19]. The main idea behind this method is to divide the image’s histogram into different categories using multiple thresholds, calculate the inter-class variance for each category, and then sum these variances. The Otsu method asserts that the optimal segmentation threshold corresponds to the point where the sum of the inter-class variances is maximized, which results in the best segmentation effect. For color images, this variance maximization is performed separately for each of the R, G, and B channels. The specific formula for the Otsu method is as follows:

$$\sigma_{B}^{2}(th_{1},th_{2},\ldots ,th_{n})=\sigma_{0}^{2}+\sigma_{1}^{2}+\ldots +\sigma_{n}^{2}$$ (24)

where:

$$\sigma_{0}^{2}=\omega_{0}(\mu_{0}-\mu_{T}{)}^{2},\;\omega_{0}=\sum_{i=0}^{t_{1}-1}p_{i},\;\mu_{0}=\sum_{i=0}^{t_{1}-1}\frac{ip_{i}}{\omega_{0}}\;\sigma_{1}^{2}=\omega_{1}(\mu_{1}-\mu_{T}{)}^{2},\;\omega_{1}=\sum_{i=t_{1}}^{t_{2}-1}p_{i},\;\mu_{1}=\sum_{i=t_{1}}^{t_{2}-1}\frac{ip_{i}}{\omega_{1}}\;\sigma_{2}^{2}=\omega_{2}(\mu_{2}-\mu_{T}{)}^{2},\;\omega_{2}=\sum_{i=t_{2}}^{t_{3}-1}p_{i},\;\mu_{2}=\sum_{i=t_{2}}^{t_{3}-1}\frac{ip_{i}}{\omega_{2}}\;\vdots \;\sigma_{n}^{2}=\omega_{n}(\mu_{n}-\mu_{T}{)}^{2},\;\omega_{n}=\sum_{i=t_{n}}^{t-1}p_{i},\;\mu_{n}=\sum_{i=t_{n}}^{t-1}\frac{ip_{i}}{\omega_{n}}$$ (25)

In the above formula, $\mu_{t}$ represents the average grayscale level of the image.

### 4.2 Kapur entropy method (maximum entropy method)

The Kapur entropy method was proposed by Kapur et al. in 1985 [18]. This method divides the histogram of an image into different categories using multiple thresholds and seeks to maximize the sum of the entropies of these categories. Assuming the image, denoted as Image, consists of *N* pixels, the threshold set is $\left[th_{1},th_{2},th_{3},\ldots ,th_{n}\right]$, which divides the image into *n* + 1 parts. The probabilities of each part are denoted as $w_{0},w_{1},w_{2},\ldots ,w_{n}$.

The specific formula for the Kapur entropy method is as follows:

$$H_{0}=-\sum_{i=0}^{th_{1}-1}\frac{p_{i}}{\omega_{0}}\ln\frac{p_{i}}{\omega_{0}},\;\omega_{0}=\sum_{i=0}^{t_{1}-1}p_{i}\;H_{1}=-\sum_{i=th_{1}}^{th_{2}-1}\frac{p_{i}}{\omega_{1}}\ln\frac{p_{i}}{\omega_{1}},\;\omega_{1}=\sum_{i=t_{1}}^{t_{2}-1}p_{i}\;H_{2}=-\sum_{i=th_{2}}^{th_{3}-1}\frac{p_{i}}{\omega_{2}}\ln\frac{p_{i}}{\omega_{2}},\;\omega_{2}=\sum_{i=t_{2}}^{t_{3}-1}p_{i}\;\vdots \;H_{n}=-\sum_{i=th_{n}}^{L-1}\frac{p_{i}}{\omega_{n}}\ln\frac{p_{i}}{\omega_{n}},\;\omega_{n}=\sum_{i=t_{n}}^{L-1}p_{i}$$ (26)

In the above formula, $H_{0},H_{1},\ldots ,H_{n}$ represent the entropy of the different classes, and *p*_*i*_ represents the probability of the *i*-th gray level. The formula is given in Formula (27), where *h*_*i*_ denotes the sum of the number of pixels at the *i*-th gray level.

$$p_{i}=\frac{h_{i}}{N},\;0\leq i\leq (L-1)$$ (27)

## 5 Analysis and discussion of experimental results

In order to comprehensively evaluate the performance of MSROA, this section selects two distinct test functions from CEC 2017 and CEC 2020 for testing its optimization performance. To highlight the superiority of MSROA, seven well-performing algorithms are chosen for comparison, including the Remora Optimization Algorithm (ROA) [50], Sand Cat Swarm Optimization (SCSO) [54], Whale Optimization Algorithm (WOA) [36], Genetic Algorithm (GA) [30], Exponential-Trigonometric Optimization (ETO) [55], Arithmetic Optimization Algorithm (AOA) [50],Prairie Dog Optimization Algorithm (PDO) [56],Particle Swarm Optimization (PSO) [33] and Crayfish Optimization Algorithm (COA) [57]. The parameter settings for these algorithms are summarized in Table 1.

**Table 1:** Parameter design of each algorithm.

Algorithm	Parameters	Value
ROA/MSROA	*C*	0.1
	*SM*	2
SCSO	Roulette Wheel selection	[0,360]
WOA	Coefficient vectors *A*	1
	Coefficient vectors *C*	[–1,1]
	Helical parameter *b*	0.75
	Helical parameter *l*	[–1,1]
GA	Crossover rate	0.7
	mutation rate	0.01
ETO	*b*	1.55
AOA	MOP_Max	1
	MOP_Min	0.2
	*A*	5
PDO	Food source alarm *ρ*	0.1
	Food source quality *ε*	2.2204*e*–16
	Individual PD difference Δ	0.005
PSO	*Wmax* *Wmin*	0.9 0.2
	*C*_1_	2
	*C*_2_	2
	*C*_*r*_	0.2
COA	*C*_*g*_	3
	*μ*	25
	*σ*	3

All experiments were conducted on a 64-bit Windows 11 operating system, using an Intel(R) Core(TM) i7-11700 processor (11th generation) with 16 GB of RAM. The simulations were executed using MATLAB R2025a. The population size *N* is 30, and the maximum evaluation count *MaxFEs* is 10000×*dim*.

### 5.1 Experimental results and analysis of CEC 2017 and CEC 2020 test functions

#### 5.1.1 Detailed description of CEC 2017 and CEC 2020 test functions.

CEC 2017 consists of 29 test functions, categorized as follows: CEC1-CEC3 are unimodal functions, CEC4-CEC10 are multimodal functions, CEC11-CEC20 are hybrid functions, and CEC21-CEC30 are composition functions. A detailed description of the CEC 2017 functions is provided in Table 2.

**Table 2:** A detailed description of CEC 2017.

Type	No.	Functions	*fmin*	Domain
Unimodal function	CEC1	Shifted and rotated bent cigar function	100	[–100, 100]
	CEC2	Shifted and rotated Zakharov’s function	300	[–100, 100]
Multimodal functions	CEC3	Shifted and rotated Rosenbrock’s function	400	[–100, 100]
	CEC4	Shifted and rotated Rastrigin’s function	500	[–100, 100]
	CEC5	Shifted and rotated expanded Schaffer’s function	600	[–100, 100]
	CEC6	Shifted and rotated Lunacek Bi_Rastrigin function	700	[–100, 100]
	CEC7	Shifted and rotated noncontinuous Rastrigin’s function	800	[–100, 100]
	CEC8	Shifted and rotated Levy function	900	[–100, 100]
	CEC9	Shifted and rotated Schwefel’s function	1000	[–100, 100]
Hybrid functions	CEC10	Hybrid function of Zakharov, Rosenbrock, and Rastrigin	1100	[–100, 100]
	CEC11	Hybrid function of high conditioned elliptic, modified Schwefel, and bent cigar	1200	[–100, 100]
	CEC12	Hybrid function of bent cigar, Rosenbrock, and Lunacek bi-Rastrigin	1300	[–100, 100]
	CEC13	Hybrid function of elliptic, Ackley, Schaffer and Rastrigin	1400	[–100, 100]
	CEC14	Hybrid function of bent cigar, HGBat, Rastrigin, and Rosenbrock	1500	[–100, 100]
	CEC15	Hybrid function of expanded Schaffer, HGBat, Rosenbrock, and modified Schwefel	1600	[–100, 100]
	CEC16	Hybrid function of Katsuura, Ackley, expanded Griewank plus Rosenbrock, modifiedSchwefel, and Rastrigin	1700	[–100, 100]
	CEC17	Hybrid function of high conditioned elliptic, Ackley, Rastrigin, HGBat, and Discus	1800	[–100, 100]
	CEC18	Hybrid function of bent cigar, Rastrigin, expanded Griewank plus Rosenbrock, Weierstrass, and expanded Schaffer	1900	[–100, 100]
	CEC19	Hybrid function of HappyCat, Katsuura, Ackley, Rastrigin, modified Schwefel, and Schaffer	2000	[–100, 100]
Composition functions	CEC20	Composition function of Rosenbrock, high conditioned elliptic, and Rastrigin	2100	[–100, 100]
	CEC21	Composition function of Rastrigin’s, Griewank, and modified Schwefel	2200	[–100, 100]
	CEC22	Composition function of Rosenbrock, Ackley, modified Schwefel, and Rastrigin	2300	[–100, 100]
	CEC23	Composition function of Ackley, high c onditioned elliptic, Griewank, and Rastrigin	2400	[–100, 100]
	CEC24	Composition function of Rastrigin, HappyCat, Ackley, Discus, and Rosenbrock	2500	[–100, 100]
	CEC25	Composition function of expanded Schaffer, modified Schwefel, Griewank, Rosenbrock,and Rastrigin	2600	[–100, 100]
	CEC26	Composition function of HGBat, Rastrigin, modified Schwefel, bent cigar, highconditioned elliptic, and expanded Schaffer	2700	[–100, 100]
	CEC27	Composition function of Ackley, Griewank, Discus, Rosenbrock, HappyCat, andexpanded Schaffer	2800	[–100, 100]
	CEC28	Composition function of shifted and rotated Rastrigin, expanded Schaffer, and Lunacek Bi_Rastrigin	2900	[–100, 100]
	CEC29	F30 composition function of shifted and rotated Rastrigin, noncontinuous Rastrigin, and Levy function	3000	[–100, 100]

CEC 2020 includes a total of 10 test functions. The CEC1-CEC4 functions are the translation-rotation function, translation-rotation Schwefel function, translation-rotation Lunacek double grating function, and extended Rosenbrock’s plus grievance function. Functions CEC5-CEC7 are mixed functions, while CEC8-CEC10 are composite functions. A detailed description of the CEC 2020 functions is shown in Table 3.

**Table 3:** A detailed description of CEC 2020.

Type	No.	Function	*fmin*	Range
Unimodal function	CEC1	Shifted and rotated bent cigar function	100	[–100, 100]
	CEC2	Shifted and rotated Schwefel’s function	1100	[–100, 100]
Simple multimodal functions	CEC3	Shifted and rotated Lunacek bi-Rastrigin function	700	[–100, 100]
	CEC4	Expanded Rosenbrock’s plus Griewangk’s function	1900	[–100, 100]
Hybrid functions	CEC5	Hybrid function 1(N=3)	1700	[–100, 100]
	CEC6	Hybrid function 2(N=4)	1600	[–100, 100]
	CEC7	Hybrid function 3(N=5)	2100	[–100, 100]
Composition functions	CEC8	Composition function 1(N=3)	2200	[–100, 100]
	CEC9	Composition function 2(N=4)	2400	[–100, 100]
	CEC10	Composition function 3(N=5)	2500	[–100, 100]

#### 5.1.2 Experimental results and analysis of CEC2017 and CEC2020 test functions.

**Fig 3:**
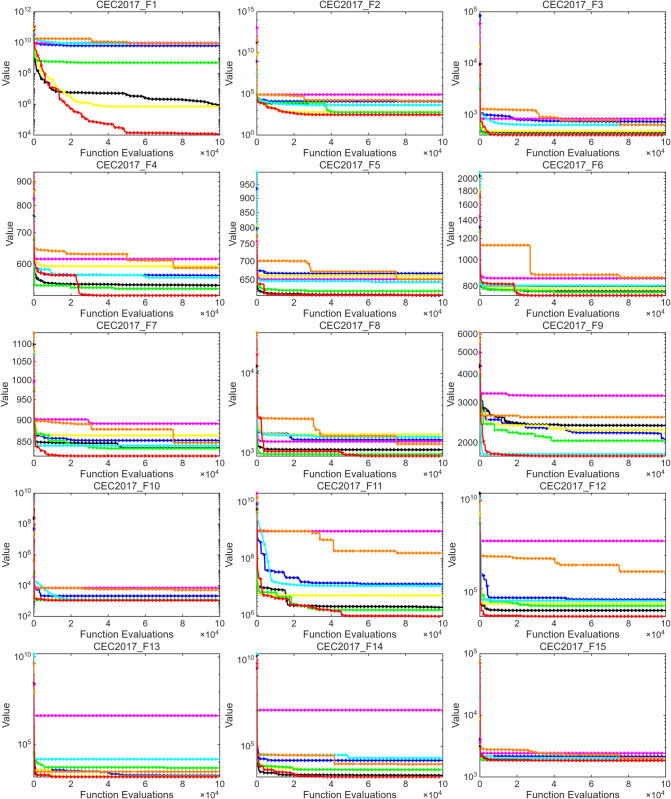
CEC2017 standard test function optimization algorithm convergence curve (F1–F15).

**Fig 4:**
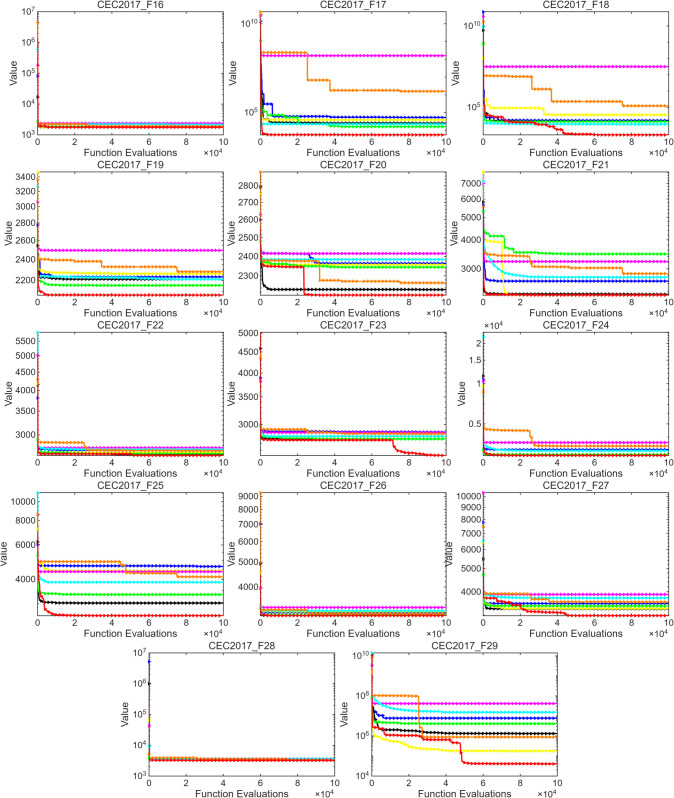
CEC2017 standard test function optimization algorithm convergence curve(F16-F29).

This section further evaluates the performance of MSROA using two benchmark test suites: CEC2017 and CEC2020. The experimental results for CEC2017 and CEC2020 are presented in Tables 4 and 5, respectively, where the optimal values are rounded for clarity. The convergence curves for the two function sets are illustrated in Figs 3 and 4.

**Table 4:** Experimental results of CEC 2017 standard test function.

CEC2017	Metric	MSROA	ROA	SCSO	WOA	GA	ETO	AOA	PDO
F1	MIN	**1.75E+03**	2.36E+09	6.78E+03	5.22E+04	3.38E+09	9.95E+06	1.33E+09	3.95E+09
	MEAN	**1.47E+04**	5.39E+09	2.40E+08	3.36E+05	6.05E+09	1.21E+08	3.96E+09	6.63E+09
	STD	**9.15E+03**	2.31E+09	6.68E+08	9.57E+05	2.28E+09	1.78E+08	2.43E+09	2.42E+09
F2	MIN	**3.00E+02**	8.00E+03	4.78E+02	4.17E+02	2.42E+04	4.12E+02	2.98E+03	6.21E+03
	MEAN	**3.01E+02**	1.22E+04	2.01E+03	9.34E+02	4.26E+04	3.18E+03	4.82E+03	9.30E+03
	STD	**4.17E-01**	2.80E+03	1.64E+03	8.98E+02	1.70E+04	2.86E+03	1.85E+03	2.81E+03
F3	MIN	**4.00E+02**	5.40E+02	4.07E+02	4.05E+02	5.97E+02	4.07E+02	4.72E+02	5.80E+02
	MEAN	**4.04E+02**	7.32E+02	4.43E+02	4.28E+02	8.67E+02	4.37E+02	5.58E+02	8.24E+02
	STD	**2.60E+00**	2.47E+02	3.12E+01	3.27E+01	3.23E+02	3.16E+01	1.22E+02	2.46E+02
F4	MIN	**5.12E+02**	5.48E+02	5.29E+02	5.40E+02	5.70E+02	5.26E+02	5.41E+02	5.68E+02
	MEAN	**5.34E+02**	5.77E+02	5.40E+02	5.56E+02	5.89E+02	5.36E+02	5.58E+02	5.82E+02
	STD	1.25E+01	2.22E+01	9.84E+00	1.57E+01	1.44E+01	**8.51E+00**	1.36E+01	1.25E+01
F5	MIN	**6.06E+02**	6.30E+02	6.07E+02	6.24E+02	6.32E+02	6.07E+02	6.36E+02	6.37E+02
	MEAN	**6.14E+02**	6.42E+02	6.17E+02	6.39E+02	6.41E+02	6.15E+02	6.43E+02	6.46E+02
	STD	5.94E+00	9.13E+00	6.94E+00	1.20E+01	7.67E+00	7.75E+00	**5.37E+00**	6.08E+00
F6	MIN	**7.29E+02**	7.93E+02	7.53E+02	7.59E+02	8.04E+02	7.37E+02	7.93E+02	7.96E+02
	MEAN	7.51E+02	8.24E+02	7.70E+02	7.83E+02	8.23E+02	**7.48E+02**	8.07E+02	8.25E+02
	STD	1.11E+01	1.97E+01	1.45E+01	1.83E+01	1.21E+01	8.39E+00	**7.82E+00**	2.72E+01
F7	MIN	**8.14E+02**	8.42E+02	8.22E+02	8.31E+02	8.60E+02	8.20E+02	8.23E+02	8.39E+02
	MEAN	8.28E+02	8.53E+02	8.32E+02	8.49E+02	8.75E+02	**8.27E+02**	8.31E+02	8.51E+02
	STD	5.60E+00	7.50E+00	6.59E+00	1.58E+01	9.33E+00	6.05E+00	**4.03E+00**	8.44E+00
F8	MIN	**9.03E+02**	1.34E+03	9.32E+02	1.09E+03	1.29E+03	9.25E+02	1.25E+03	1.20E+03
	MEAN	**9.55E+02**	1.63E+03	1.04E+03	1.35E+03	1.61E+03	1.06E+03	1.49E+03	1.48E+03
	STD	**4.99E+01**	2.47E+02	1.07E+02	2.54E+02	3.23E+02	1.10E+02	1.64E+02	2.35E+02
F9	MIN	**1.25E+03**	2.19E+03	1.71E+03	1.92E+03	2.73E+03	1.87E+03	1.95E+03	2.09E+03
	MEAN	**1.79E+03**	2.52E+03	1.97E+03	2.19E+03	3.01E+03	2.08E+03	2.18E+03	2.44E+03
	STD	2.88E+02	1.93E+02	1.78E+02	2.59E+02	1.93E+02	2.02E+02	**1.69E+02**	1.78E+02
F10	MIN	**1.11E+03**	1.22E+03	1.13E+03	1.16E+03	2.17E+03	1.12E+03	1.14E+03	2.44E+03
	MEAN	**1.14E+03**	1.71E+03	1.16E+03	1.24E+03	1.48E+04	1.15E+03	1.17E+03	3.60E+03
	STD	**1.52E+01**	9.50E+02	4.62E+01	8.80E+01	1.91E+04	5.18E+01	3.09E+01	8.87E+02
F11	MIN	**4.08E+03**	3.46E+06	1.17E+05	5.22E+05	2.30E+08	1.97E+05	1.36E+05	3.76E+07
	MEAN	1.13E+06	5.29E+07	9.45E+05	5.60E+06	5.01E+08	**7.08E+05**	1.81E+06	1.25E+08
	STD	1.11E+06	1.29E+08	8.19E+05	5.16E+06	2.72E+08	**4.89E+05**	2.12E+06	9.21E+07
F12	MIN	**2.18E+03**	5.50E+03	5.48E+03	1.05E+04	1.22E+07	4.40E+03	6.75E+03	2.13E+04
	MEAN	**8.97E+03**	1.54E+04	1.40E+04	2.56E+04	7.26E+07	1.32E+04	1.18E+04	9.84E+05
	STD	**5.03E+03**	8.60E+03	7.46E+03	1.40E+04	6.83E+07	7.23E+03	6.69E+03	1.20E+06
F13	MIN	**1.43E+03**	1.53E+03	1.47E+03	1.50E+03	2.53E+05	1.47E+03	2.11E+03	1.96E+03
	MEAN	**1.46E+03**	2.67E+03	2.81E+03	1.68E+03	5.75E+06	2.80E+03	1.01E+04	5.92E+03
	STD	**1.91E+01**	1.45E+03	1.77E+03	6.57E+02	9.93E+06	1.71E+03	8.11E+03	5.63E+03
F14	MIN	**1.55E+03**	5.64E+03	1.69E+03	2.27E+03	9.76E+04	1.76E+03	8.41E+03	3.17E+03
	MEAN	**1.64E+03**	1.05E+04	3.01E+03	4.66E+03	5.49E+06	4.38E+03	1.35E+04	6.37E+03
	STD	**5.55E+01**	3.92E+03	1.74E+03	2.08E+03	6.83E+06	2.06E+03	4.23E+03	2.83E+03
F15	MIN	**1.60E+03**	1.81E+03	1.66E+03	1.75E+03	2.23E+03	1.73E+03	1.93E+03	1.96E+03
	MEAN	**1.69E+03**	1.99E+03	1.84E+03	1.88E+03	2.43E+03	1.88E+03	2.07E+03	2.06E+03
	STD	**5.90E+01**	1.25E+02	1.07E+02	1.05E+02	1.51E+02	1.20E+02	1.03E+02	7.09E+01
F16	MIN	**1.73E+03**	1.78E+03	1.75E+03	1.76E+03	2.04E+03	1.74E+03	1.79E+03	1.79E+03
	MEAN	**1.76E+03**	1.85E+03	1.77E+03	1.81E+03	2.18E+03	1.78E+03	1.90E+03	1.83E+03
	STD	1.86E+01	5.90E+01	**1.71E+01**	4.99E+01	1.05E+02	4.76E+01	8.57E+01	4.65E+01
F17	MIN	**3.41E+03**	8.24E+03	7.08E+03	5.27E+03	9.48E+06	9.45E+03	6.24E+03	5.54E+04
	MEAN	**1.28E+04**	2.58E+04	2.82E+04	2.01E+04	1.14E+08	2.24E+04	1.72E+04	2.92E+06
	STD	8.72E+03	1.30E+04	1.31E+04	1.28E+04	1.16E+08	1.07E+04	**7.91E+03**	4.14E+06
F18	MIN	1.94E+03	3.69E+03	**1.92E+03**	7.37E+03	8.39E+05	1.93E+03	2.91E+03	2.84E+04
	MEAN	**2.10E+03**	1.63E+05	1.35E+04	6.58E+04	4.90E+07	5.80E+03	2.70E+04	1.80E+05
	STD	**1.53E+02**	6.10E+05	4.72E+04	1.27E+05	1.37E+08	5.11E+03	1.89E+04	1.84E+05
F19	MIN	**2.03E+03**	2.10E+03	2.05E+03	2.11E+03	2.25E+03	2.04E+03	2.08E+03	2.24E+03
	MEAN	**2.07E+03**	2.20E+03	2.13E+03	2.19E+03	2.38E+03	2.14E+03	2.16E+03	2.29E+03
	STD	**2.32E+01**	6.28E+01	4.99E+01	6.52E+01	1.12E+02	5.79E+01	6.33E+01	4.60E+01
F20	MIN	**2.20E+03**	2.26E+03	2.21E+03	2.32E+03	2.37E+03	2.32E+03	2.32E+03	2.23E+03
	MEAN	**2.21E+03**	2.33E+03	2.30E+03	2.35E+03	2.39E+03	2.34E+03	2.34E+03	2.27E+03
	STD	**2.47E+00**	3.35E+01	4.97E+01	1.27E+01	1.71E+01	1.00E+01	1.56E+01	4.89E+01
F21	MIN	**2.25E+03**	2.40E+03	2.30E+03	2.31E+03	2.73E+03	2.31E+03	2.50E+03	2.70E+03
	MEAN	**2.31E+03**	2.74E+03	2.33E+03	2.50E+03	3.00E+03	2.37E+03	2.73E+03	2.85E+03
	STD	**1.21E+01**	3.44E+02	2.74E+01	4.37E+02	3.93E+02	1.42E+02	2.26E+02	1.05E+02
F22	MIN	**2.61E+03**	2.64E+03	2.63E+03	2.63E+03	2.69E+03	2.63E+03	2.69E+03	2.68E+03
	MEAN	**2.63E+03**	2.68E+03	2.65E+03	2.65E+03	2.72E+03	2.65E+03	2.73E+03	2.70E+03
	STD	1.10E+01	3.39E+01	**8.55E+00**	1.41E+01	1.93E+01	1.22E+01	3.80E+01	1.02E+01
F23	MIN	**2.50E+03**	2.78E+03	2.55E+03	2.75E+03	2.83E+03	2.77E+03	2.79E+03	2.81E+03
	MEAN	**2.63E+03**	2.83E+03	2.75E+03	2.78E+03	2.86E+03	2.79E+03	2.87E+03	2.83E+03
	STD	1.15E+02	5.12E+01	5.47E+01	1.85E+01	1.91E+01	**1.24E+01**	5.04E+01	1.36E+01
F24	MIN	**2.90E+03**	3.01E+03	2.92E+03	2.95E+03	3.12E+03	2.91E+03	3.01E+03	3.13E+03
	MEAN	**2.92E+03**	3.17E+03	2.95E+03	2.97E+03	3.27E+03	2.95E+03	3.07E+03	3.21E+03
	STD	**2.20E+01**	1.51E+02	2.93E+01	2.82E+01	1.05E+02	3.10E+01	5.96E+01	7.73E+01
F25	MIN	**2.80E+03**	3.34E+03	2.90E+03	3.08E+03	3.67E+03	2.98E+03	3.45E+03	3.47E+03
	MEAN	**2.90E+03**	3.91E+03	3.05E+03	3.73E+03	4.16E+03	3.39E+03	4.00E+03	3.87E+03
	STD	**6.27E+01**	3.80E+02	9.34E+01	5.30E+02	3.19E+02	4.36E+02	3.42E+02	2.70E+02
F26	MIN	**3.09E+03**	3.13E+03	3.10E+03	3.10E+03	3.18E+03	3.10E+03	3.18E+03	3.12E+03
	MEAN	**3.10E+03**	3.16E+03	3.11E+03	3.14E+03	3.24E+03	3.13E+03	3.26E+03	3.13E+03
	STD	**2.86E+00**	3.24E+01	2.33E+01	3.68E+01	5.97E+01	3.38E+01	6.69E+01	7.11E+00
F27	MIN	**3.10E+03**	3.43E+03	3.22E+03	3.40E+03	3.65E+03	3.21E+03	3.47E+03	3.43E+03
	MEAN	**3.26E+03**	3.62E+03	3.35E+03	3.50E+03	3.81E+03	3.36E+03	3.69E+03	3.54E+03
	STD	1.44E+02	1.39E+02	9.55E+01	1.44E+02	1.20E+02	1.17E+02	1.04E+02	**7.56E+01**
F28	MIN	**3.16E+03**	3.29E+03	3.17E+03	3.27E+03	3.42E+03	3.19E+03	3.27E+03	3.26E+03
	MEAN	**3.22E+03**	3.37E+03	3.24E+03	3.36E+03	3.55E+03	3.25E+03	3.41E+03	3.34E+03
	STD	**3.39E+01**	9.12E+01	5.43E+01	7.08E+01	1.05E+02	5.44E+01	1.16E+02	7.07E+01
F29	MIN	**6.66E+03**	8.85E+05	1.30E+04	4.50E+04	1.14E+07	7.13E+04	1.67E+06	8.86E+05
	MEAN	**1.42E+05**	7.25E+06	6.37E+05	8.89E+05	2.49E+07	1.50E+06	9.60E+06	9.53E+05
	STD	**2.13E+05**	8.23E+06	7.55E+05	1.19E+06	9.55E+06	2.47E+06	9.57E+06	2.64E+05

**Table 5:** Experimental results of CEC 2020 standard test function.

CEC2020	Metric	MSROA	ROA	SCSO	WOA	GA	ETO	AOA	PDO
F1	MIN	**2.24E+03**	1.98E+09	4.29E+03	6.21E+04	3.78E+09	1.18E+07	1.38E+09	3.85E+09
	MEAN	**1.43E+04**	4.64E+09	7.91E+07	5.48E+05	6.63E+09	1.24E+08	3.56E+09	5.74E+09
	STD	**8.31E+03**	3.21E+09	1.53E+08	8.47E+05	2.37E+09	1.87E+08	1.89E+09	1.39E+09
F2	MIN	**1.54E+03**	1.99E+03	1.80E+03	1.89E+03	2.69E+03	1.64E+03	1.88E+03	2.23E+03
	MEAN	**1.90E+03**	2.36E+03	2.10E+03	2.23E+03	3.03E+03	1.92E+03	2.19E+03	2.49E+03
	STD	1.84E+02	2.51E+02	1.89E+02	2.46E+02	2.17E+02	1.90E+02	2.03E+02	**1.69E+02**
F3	MIN	**7.21E+02**	7.95E+02	7.56E+02	7.59E+02	8.06E+02	7.41E+02	7.89E+02	8.04E+02
	MEAN	**7.46E+02**	8.23E+02	7.74E+02	7.81E+02	8.29E+02	7.58E+02	8.01E+02	8.36E+02
	STD	1.27E+01	1.73E+01	1.30E+01	1.58E+01	1.25E+01	1.44E+01	**7.57E+00**	2.37E+01
F4	MIN	**1.90E+03**	**1.90E+03**	**1.90E+03**	**1.90E+03**	1.90E+03	**1.90E+03**	**1.90E+03**	**1.90E+03**
	MEAN	**1.90E+03**	**1.90E+03**	**1.90E+03**	1.90E+03	1.90E+03	1.90E+03	**1.90E+03**	**1.90E+03**
	STD	**0.00E+00**	**0.00E+00**	**0.00E+00**	7.03E-02	8.49E-08	2.70E-01	**0.00E+00**	**0.00E+00**
F5	MIN	**2.13E+03**	3.35E+04	3.87E+03	1.02E+04	1.70E+06	4.24E+03	1.87E+04	9.08E+04
	MEAN	**5.41E+03**	5.43E+05	5.24E+04	3.15E+05	1.16E+07	2.13E+04	3.78E+04	3.43E+05
	STD	**3.00E+03**	2.84E+05	1.17E+05	4.39E+05	9.51E+06	4.89E+04	1.93E+04	1.69E+05
F6	MIN	**1.60E+03**	1.77E+03	1.74E+03	1.75E+03	2.18E+03	1.73E+03	1.84E+03	1.93E+03
	MEAN	**1.72E+03**	1.95E+03	1.82E+03	1.86E+03	2.39E+03	1.84E+03	1.99E+03	1.97E+03
	STD	6.08E+01	1.24E+02	6.11E+01	7.61E+01	1.34E+02	1.06E+02	1.19E+02	**3.97E+01**
F7	MIN	**2.59E+03**	4.25E+03	3.52E+03	6.92E+03	9.91E+05	3.34E+03	4.84E+03	3.82E+04
	MEAN	**5.02E+03**	1.26E+05	9.54E+03	2.13E+04	7.70E+06	9.80E+03	7.20E+03	2.58E+05
	STD	1.93E+03	6.13E+05	3.39E+03	1.47E+04	1.34E+07	3.81E+03	**1.87E+03**	4.54E+05
F8	MIN	**2.23E+03**	2.42E+03	2.30E+03	2.31E+03	2.61E+03	2.31E+03	2.54E+03	2.68E+03
	MEAN	**2.30E+03**	2.65E+03	2.33E+03	2.48E+03	2.83E+03	2.39E+03	2.76E+03	2.85E+03
	STD	**2.17E+01**	2.74E+02	3.39E+01	3.93E+02	1.73E+02	2.47E+02	2.18E+02	1.28E+02
F9	MIN	**2.50E+03**	2.78E+03	2.74E+03	2.76E+03	2.83E+03	2.76E+03	2.82E+03	2.81E+03
	MEAN	**2.62E+03**	2.82E+03	2.77E+03	2.79E+03	2.85E+03	2.78E+03	2.87E+03	2.83E+03
	STD	1.14E+02	3.55E+01	1.57E+01	2.39E+01	2.26E+01	1.77E+01	3.63E+01	**1.37E+01**
F10	MIN	**2.60E+03**	3.02E+03	2.92E+03	2.95E+03	3.12E+03	2.92E+03	3.01E+03	3.12E+03
	MEAN	**2.89E+03**	3.19E+03	2.96E+03	2.96E+03	3.24E+03	2.96E+03	3.07E+03	3.22E+03
	STD	1.00E+02	1.29E+02	2.16E+01	**7.34E+00**	1.14E+02	3.05E+01	3.60E+01	6.42E+01

Overall, for CEC2017,MSROA demonstrates the best performance among all compared algorithms. For most test functions, such as F1–F3, F8–F10, and F12–F16, its MIN and MEAN values are significantly lower than those of other algorithms. For instance, the MIN value for F1 is only 1.75 × 10^3^, which is considerably better than that of ROA (2.36 × 10^9^). Moreover, MSROA exhibits relatively small STD values, indicating strong stability and low result variability. In contrast, algorithms such as GA, ROA, and PDO perform poorly. GA yields extremely high values in functions like F10, F11, and F29 (MEAN of 1.48 × 10^4^ for F10), along with excessively large STD values, reflecting weak robustness. Similarly, ROA and PDO result in high MIN and MEAN values across many functions, demonstrating subpar optimization capability. The SCSO, WOA, ETO, and AOA algorithms perform moderately. While they achieve good results in certain functions (F18 and F22), their overall performance is less stable and efficient than that of MSROA. As shown in Figs 3 and 4, MSROA consistently finds the optimal solution across most test functions and converges more rapidly than the compared algorithms. For unimodal functions such as F1 and F2, MSROA exhibits notably faster convergence, requiring fewer function evaluations to reach the optimum. For multimodal functions like F4, F6, and F8, MSROA demonstrates the ability to escape local optima during the middle and late stages of evolution. In the case of composite functions, MSROA also exhibits superior global search capability compared to other methods. This performance can be attributed to the restart mechanism embedded in MSROA. In functions such as F23, F27, and F29, this mechanism enables the algorithm to escape from local optima and locate the global optimum effectively.

Table 5 compares the performance of several optimization algorithms, including MSROA and ROA, on functions F1–F10 from the CEC2020 test set. The evaluation includes three indicators: the minimum value (MIN), which reflects the best convergence achieved; the mean value (MEAN), indicating overall stability; and the standard deviation (STD), which measures the dispersion of results. MSROA exhibits outstanding performance, achieving lower optimal values in several functions (F1 and F2), while maintaining relatively controlled MEAN and STD values. This demonstrates both high convergence accuracy and stable optimization behavior. Although ROA and SCSO occasionally attain competitive optimal values, their corresponding MEAN and STD are often large and unstable, indicating significant result fluctuations and limiting their practical value in engineering applications. Special attention should be paid to function F4, where all algorithms exhibit a standard deviation of 0. We confirm that this result is not due to decimal truncation. Instead, it indicates that the topological structure of function F4 is relatively simple for the algorithms tested in this study. All participating algorithms were able to locate the theoretical global optimum in every independent run without exception. Consequently, while F4 confirms that all algorithms possess basic convergence capabilities, it does not provide a challenge sufficient to differentiate their performance.

**Fig 5:**
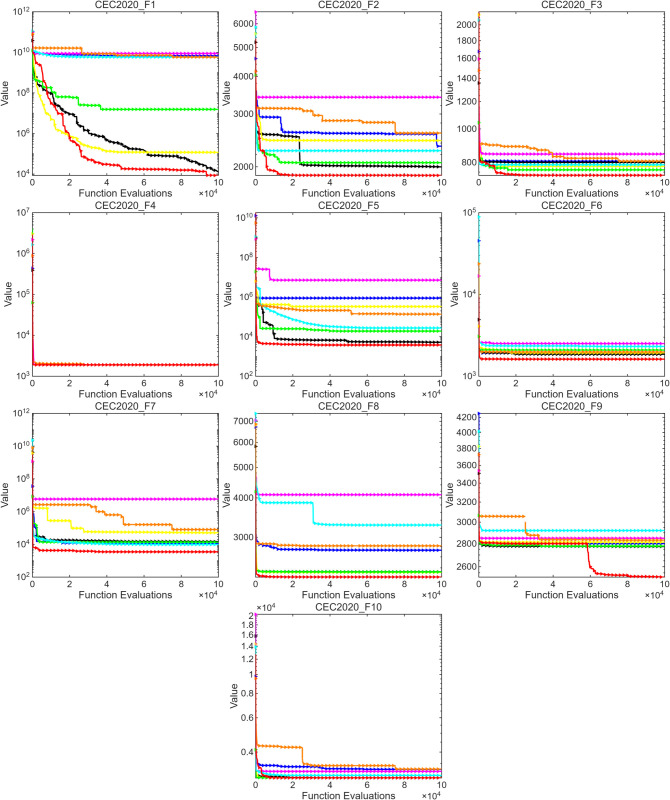
CEC2020 standard test function optimization algorithm convergence curve.

Fig 5 illustrates the convergence performance of eight algorithms, including MSROA and ROA. Overall, MSROA demonstrates the best performance across most test functions: its convergence curves are consistently lower, exhibit rapid descent, and stabilize quickly as the number of function evaluations increases, indicating fast convergence and high final accuracy. Specifically, in functions F1, F2, and F3, MSROA significantly outperforms the other algorithms. Its advantages become even more pronounced in more complex functions such as F5 and F7. In contrast, ROA and SCSO show relatively poor performance, with convergence curves generally positioned higher, exhibiting slower descent and higher final values. This is especially evident in F1, F5, and F7, where their convergence efficiency is noticeably lower than that of the other algorithms. Algorithms such as WOA and GA show moderate performance, with curve positions and convergence rates falling between those of MSROA and ROA. Although ETO, AOA, and PDO exhibit slight fluctuations, their overall performance still lags behind MSROA. For function F4, all algorithms present similar convergence behavior, confirming earlier observations that F4 is a relatively simple function. As such, it serves as a benchmark for evaluating the baseline convergence capabilities of algorithms.

In summary, MSROA exhibits superior convergence speed and accuracy across most functions, particularly in complex scenarios, while ROA and SCSO show weaker performance. Function F4 remains a useful reference for basic convergence verification.

#### 5.1.3 Analysis of Wilcoxon rank sum test results.

Table 6 presents the results of the Wilcoxon rank-sum test comparing MSROA with seven other algorithms (including ROA and SCSO) on the CEC2017 test suite across functions F1–F29. To ensure the statistical validity of the comparison, the test is performed based on the data obtained from 30 independent runs for each algorithm on every function. The *p*-value is used to evaluate the statistical significance of performance differences, where *p* < 0.05 indicates a significant difference. The last row, denoted by ‘ + / −/  = ’, represents the number of functions where MSROA performs significantly better, worse, or has no significant difference, respectively. Overall, MSROA demonstrates a clear advantage. Compared to ROA, GA, and PDO, the results are ‘29/0/0’, indicating that MSROA significantly outperforms these algorithms on all 29 functions. Against WOA, the result is ‘28/0/1’, with only one function showing no significant difference, highlighting MSROA’s nearly comprehensive superiority. For algorithms with more competitive performance, the differences are more nuanced. Compared to SCSO, MSROA achieves ‘19/0/10’, suggesting significant improvements in 19 functions and no significant difference in the remaining 10. Similar trends are observed for ETO (‘20/0/9’) and AOA (‘25/0/4’). In most cases, *p*-values are far below 0.05, reinforcing the reliability of the observed superiority. Only a few cases (SCSO on F4 and F5) show p≥0.05, implying comparable performance on these specific functions.

**Table 6:** The Wilcoxon rank sum test data of the CEC 2017 test function.

CEC2017	MSROA VS ROA	MSROA VS SCSO	MSROA VS WOA	MSROA VS GA	MSROA VS ETO	MSROA VS AOA	MSROA VS PDO
F1	1.73E-06	7.69E-06	1.73E-06	1.73E-06	1.73E-06	1.73E-06	1.73E-06
F2	1.73E-06	1.73E-06	1.73E-06	1.73E-06	1.73E-06	1.73E-06	1.73E-06
F3	1.73E-06	1.73E-06	2.61E-04	1.73E-06	1.92E-06	1.73E-06	1.73E-06
F4	3.18E-06	**1.36E-01**	3.88E-04	1.73E-06	**6.73E-01**	1.15E-04	1.73E-06
F5	1.73E-06	**2.37E-01**	1.73E-06	1.73E-06	**8.13E-01**	1.73E-06	1.73E-06
F6	1.73E-06	1.29E-03	5.75E-06	1.73E-06	**2.80E-01**	1.73E-06	1.73E-06
F7	1.73E-06	**1.02E-01**	6.34E-06	1.73E-06	**4.78E-01**	**8.59E-02**	1.73E-06
F8	1.73E-06	6.42E-03	1.73E-06	1.73E-06	2.96E-03	1.73E-06	1.73E-06
F9	1.73E-06	**8.59E-02**	1.11E-03	1.73E-06	1.57E-02	3.06E-04	1.92E-06
F10	1.73E-06	**2.37E-01**	2.88E-06	1.73E-06	**9.43E-01**	2.26E-03	1.73E-06
F11	1.73E-06	**6.14E-01**	1.96E-03	1.73E-06	**2.21E-01**	**4.17E-01**	1.73E-06
F12	3.33E-02	**8.22E-02**	2.22E-04	1.73E-06	**8.97E-02**	**4.28E-01**	1.73E-06
F13	1.73E-06	6.32E-05	1.73E-06	1.73E-06	2.41E-04	1.73E-06	1.73E-06
F14	1.73E-06	2.88E-06	1.73E-06	1.73E-06	1.73E-06	1.73E-06	1.73E-06
F15	1.73E-06	3.32E-04	3.88E-06	1.73E-06	4.45E-05	1.73E-06	1.73E-06
F16	5.22E-06	**2.13E-01**	4.20E-04	1.73E-06	**4.28E-01**	1.92E-06	2.35E-06
F17	4.11E-03	1.20E-03	**6.87E-02**	1.73E-06	1.25E-02	**1.59E-01**	1.73E-06
F18	1.73E-06	**6.29E-01**	1.73E-06	1.73E-06	4.07E-02	1.73E-06	1.73E-06
F19	2.13E-06	6.16E-04	2.13E-06	1.73E-06	1.36E-04	4.07E-05	1.73E-06
F20	1.73E-06	2.13E-06	1.73E-06	1.73E-06	1.73E-06	1.73E-06	1.73E-06
F21	1.73E-06	1.04E-02	1.48E-03	1.73E-06	1.38E-03	1.73E-06	1.73E-06
F22	1.24E-05	8.94E-04	4.53E-04	1.73E-06	1.29E-03	1.73E-06	1.73E-06
F23	1.73E-06	4.53E-04	2.60E-06	1.73E-06	1.73E-06	1.73E-06	1.73E-06
F24	1.73E-06	6.16E-04	1.73E-06	1.73E-06	3.88E-04	1.73E-06	1.73E-06
F25	1.73E-06	2.60E-05	1.73E-06	1.73E-06	4.29E-06	1.73E-06	1.73E-06
F26	1.73E-06	1.59E-03	1.73E-06	1.73E-06	4.73E-06	1.73E-06	1.73E-06
F27	1.73E-06	1.96E-02	4.20E-04	1.73E-06	2.07E-02	1.73E-06	1.73E-06
F28	1.73E-06	**2.37E-01**	1.92E-06	1.73E-06	**7.19E-02**	1.92E-06	2.60E-06
F29	1.73E-06	4.28E-02	2.83E-04	1.73E-06	3.06E-04	1.73E-06	1.73E-06
+/-/=	29/0/0	19/0/10	28/0/1	29/0/0	20/0/9	25/0/4	29/0/0

The CEC2020 results in Table 7 further support MSROA’s advantage. Most *p*-values are significantly below 0.05—for instance, the *p*-value for MSROA vs. ROA on F1 is 1.73 × 10^−6^, indicating a substantial difference. MSROA achieves a ‘10/0/0’ result against GA, outperforming it significantly in all 10 functions. Against six other algorithms, including ROA and SCSO, MSROA obtains ‘9/0/1’, with the only exception being F4 (p≥0.05,*p* = 1.00 for ROA), again confirming that this function is relatively easy and not sufficient for distinguishing algorithmic performance.

**Table 7:** The Wilcoxon rank sum test data of CEC 2020 test function.

CEC2017	MSROA VS ROA	MSROA VS SCSO	MSROA VS WOA	MSROA VS GA	MSROA VS ETO	MSROA VS AOA	MSROA VS PDO
F1	1.73E-06	1.06E-04	1.73E-06	1.73E-06	1.73E-06	1.73E-06	1.73E-06
F2	2.84E-05	1.17E-02	1.04E-03	1.73E-06	**9.92E-01**	1.20E-03	1.73E-06
F3	1.73E-06	1.02E-05	4.29E-06	1.73E-06	3.00E-02	1.73E-06	1.73E-06
F4	**1.00E+00**	**1.00E+00**	**5.00E-01**	1.73E-06	1.32E-04	**1.00E+00**	**1.00E+00**
F5	1.73E-06	2.30E-02	2.13E-06	1.73E-06	5.67E-03	1.73E-06	1.73E-06
F6	4.73E-06	3.06E-04	1.36E-05	1.73E-06	3.32E-04	1.92E-06	1.73E-06
F7	2.05E-04	2.83E-04	3.88E-06	1.73E-06	4.53E-04	5.32E-03	1.73E-06
F8	1.73E-06	1.04E-03	1.49E-05	1.73E-06	1.97E-05	1.73E-06	1.73E-06
F9	1.73E-06	2.22E-04	2.35E-06	1.73E-06	3.52E-06	1.73E-06	1.73E-06
F10	1.73E-06	3.32E-04	1.73E-06	1.73E-06	7.16E-04	1.73E-06	1.73E-06
+/-/=	9/0/1	9/0/1	9/0/1	10/0/0	9/0/1	9/0/1	9/0/1

In summary, the Wilcoxon rank-sum test confirms that MSROA delivers statistically significant improvements over most baseline algorithms on both CEC2017 and CEC2020, especially over ROA, GA, and PDO, and is only indistinguishable from some algorithms on a limited number of simple functions.

### 5.2 Multi threshold image segmentation experiment

#### 5.2.1 Dataset and experimental parameter settings.

**Fig 6:**
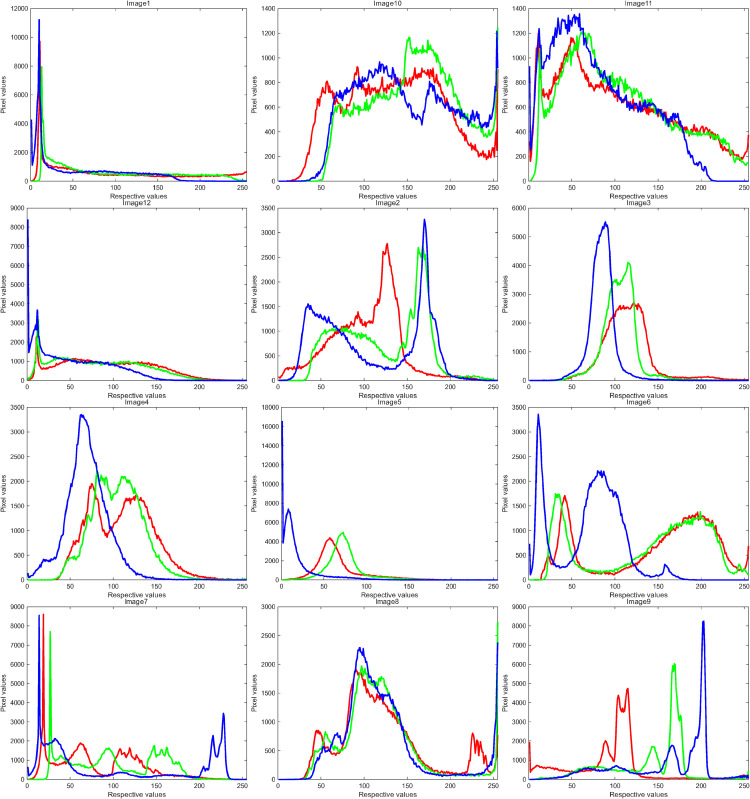
Histogram of the original image.

This section evaluates the optimization performance of the proposed MSROA algorithm in comparison with seven existing image segmentation algorithms. The experimental datasets consist of 12 images selected from the Berkeley Segmentation Dataset and Benchmark 500 (BSDS500) [58]. Fig 6 presents the original images along with the corresponding histograms of the red (R), green (G), and blue (B) color channels. To ensure fair comparison, the population size *N* is set to 30. The threshold *K* is assigned varying values corresponding to the algorithm dimension dim (15, 20, 25, and 30), and the maximum number of function evaluations is defined as maxFES=dim×3000.

#### 5.2.2 Statistical results and analysis of image segmentation.

Table 8 presents the performance of eight algorithms across 12 images and four threshold levels (K=15,20,25,30), using Otsu’s method as the fitness criterion (where a higher value indicates better segmentation quality). Overall, MSROA consistently achieves superior performance. Across all image-threshold combinations, MSROA yields high fitness values, often outperforming or matching those of comparable algorithms such as ROA and WOA. For instance, when *K* = 15 for Image 1, MSROA achieves 5322.64, slightly outperforming ROA (5321.04) and WOA (5321.99). Similarly, for Image 6 at *K* = 30, MSROA attains 5720.92, surpassing ROA (5719.62) and WOA (5720.21). Compared to lower-performing algorithms such as AOA and PDO, MSROA demonstrates significant advantages. For example, at *K* = 15 for Image 10, MSROA reaches 3982.70, considerably higher than AOA (3966.57) and PDO (3961.21); for Image 12 at *K* = 20, MSROA records 2799.22, outperforming AOA (2787.31) and PDO (2784.61). Moreover, as the threshold *K* increases, MSROA’s fitness value exhibits a steady upward trend, with consistent performance advantages across all threshold levels, highlighting its robustness under varying parameter settings. In conclusion, when Otsu’s criterion is employed as the objective function, MSROA demonstrates leading performance across most test scenarios, validating its effectiveness and reliability in image segmentation tasks.

**Table 8:** Statistical results based on Otsu method.

Images	Threshold	MSROA	ROA	SCSO	WOA	PSO	COA	AOA	PDO
1	K=15	**5322.64**	5321.04	5319.74	5321.99	5320.64	5321.80	5305.85	5302.25
	K=20	**5328.52**	5326.76	5326.30	5327.93	5327.07	5327.68	5315.92	5314.70
	K=25	**5331.37**	5329.72	5329.66	5330.76	5330.57	5330.46	5321.69	5321.25
	K=30	**5332.94**	5331.54	5331.49	5332.30	5332.25	5332.12	5325.32	5324.87
2	K=15	**2482.16**	2480.85	2478.76	2481.33	2480.23	2481.75	2468.57	2463.14
	K=20	**2486.76**	2485.44	2484.33	2485.85	2485.66	2486.09	2475.57	2472.97
	K=25	**2489.00**	2487.79	2487.18	2488.11	2488.08	2488.36	2480.05	2478.82
	K=30	**2490.31**	2489.10	2488.71	2489.57	2489.80	2489.66	2483.25	2482.52
3	K=15	**6226.12**	6223.90	6222.46	6225.25	6224.35	6225.12	6210.28	6206.51
	K=20	**6231.76**	6229.70	6229.37	6230.87	6230.36	6230.95	6219.11	6218.27
	K=25	**6234.65**	6232.87	6232.39	6233.83	6233.87	6233.70	6224.91	6224.08
	K=30	**6236.20**	6234.68	6234.30	6235.38	6235.55	6235.37	6228.52	6228.57
4	K=15	**1077.18**	1076.09	1075.48	1076.21	1075.88	1076.84	1068.10	1064.57
	K=20	**1079.81**	1078.71	1078.41	1078.83	1078.99	1079.36	1072.47	1070.98
	K=25	**1081.14**	1080.17	1079.90	1080.30	1080.46	1080.62	1074.99	1074.24
	K=30	**1081.87**	1080.99	1080.81	1081.10	1081.36	1081.34	1076.88	1076.28
5	K=15	**1358.25**	1357.51	1356.17	1357.65	1356.95	1357.81	1349.02	1345.46
	K=20	**1360.46**	1359.74	1358.87	1359.79	1359.59	1359.97	1353.02	1350.81
	K=25	**1361.58**	1360.86	1360.06	1360.91	1360.91	1361.13	1355.56	1354.03
	K=30	**1362.19**	1361.51	1361.02	1361.63	1361.64	1361.77	1356.99	1356.45
6	K=15	**5711.83**	5710.16	5708.18	5711.23	5709.59	5711.19	5697.13	5692.29
	K=20	**5716.98**	5715.28	5713.92	5716.28	5715.61	5716.17	5704.89	5703.34
	K=25	**5719.44**	5718.13	5717.20	5718.80	5718.80	5718.61	5710.25	5709.45
	K=30	**5720.92**	5719.62	5718.97	5720.21	5720.35	5720.01	5713.72	5712.95
7	K=15	**4319.72**	4318.22	4316.93	4318.91	4318.08	4319.24	4305.88	4301.96
	K=20	**4324.16**	4322.60	4321.93	4323.38	4322.73	4323.47	4313.09	4311.54
	K=25	**4326.26**	4324.95	4324.77	4325.50	4325.68	4325.57	4318.14	4316.85
	K=30	**4327.49**	4326.27	4326.21	4326.77	4326.92	4326.79	4320.47	4320.06
8	K=15	**2887.25**	2886.03	2883.01	2886.13	2885.55	2887.10	2873.92	2869.18
	K=20	**2891.99**	2890.60	2889.22	2891.33	2890.96	2891.71	2881.35	2878.73
	K=25	**2894.26**	2893.05	2892.22	2893.53	2893.65	2893.81	2885.66	2884.63
	K=30	**2895.64**	2894.36	2894.04	2894.98	2895.09	2895.10	2888.75	2888.30
9	K=15	**4626.82**	4625.09	4623.74	4625.87	4625.08	4626.52	4614.38	4610.91
	K=20	**4631.44**	4629.84	4629.29	4630.40	4630.57	4630.87	4621.78	4620.10
	K=25	**4633.65**	4632.26	4632.04	4632.64	4633.04	4633.11	4626.42	4625.42
	K=30	**4634.97**	4633.54	4633.68	4634.05	4634.52	4634.30	4628.80	4628.32
10	K=15	**3982.70**	3981.35	3977.43	3982.15	3980.79	3982.44	3966.57	3961.21
	K=20	**3988.63**	3987.13	3984.98	3987.95	3987.18	3988.03	3976.22	3973.28
	K=25	**3991.42**	3990.02	3988.49	3990.83	3990.47	3990.65	3981.52	3980.78
	K=30	**3992.99**	3991.66	3990.92	3992.29	3992.38	3992.21	3985.03	3984.92
11	K=15	**3829.13**	3827.37	3825.47	3828.28	3826.90	3828.35	3810.58	3804.56
	K=20	**3836.26**	3834.45	3833.30	3835.59	3834.39	3835.26	3822.28	3819.85
	K=25	**3839.68**	3837.93	3837.59	3838.97	3838.70	3838.67	3828.74	3828.16
	K=30	**3841.60**	3840.09	3839.87	3840.77	3840.86	3840.59	3833.22	3832.34
12	K=15	**2794.37**	2793.28	2791.24	2793.60	2791.94	2793.56	2779.92	2773.26
	K=20	**2799.22**	2798.04	2796.87	2798.45	2797.76	2798.44	2787.31	2784.61
	K=25	**2801.65**	2800.44	2799.70	2800.98	2800.67	2800.78	2792.39	2790.53
	K=30	**2803.01**	2801.94	2801.51	2802.27	2802.11	2802.20	2794.99	2794.54

Table 9 presents the performance results of MSROA and seven other algorithms across 12 images and four threshold levels (K=15,20,25,30), with Kapur’s entropy serving as the fitness function. MSROA demonstrates consistently superior performance. Across all image-threshold combinations, MSROA frequently ranks among the top performers, often achieving the highest or second-highest fitness value. For example, when *K* = 30 for Image 9, MSROA achieves a value of 63.7909, significantly outperforming ROA (54.8521), SCSO (60.9884), and others. Similarly, at *K* = 30 for Image 3, MSROA reaches 62.6638, surpassing WOA (61.5811) and PSO (61.3898). Compared to lower-performing algorithms such as AOA and PDO, MSROA shows clear advantages. For instance, at *K* = 15 for Image 5, MSROA achieves 40.7560, far exceeding AOA (36.3560) and PDO (35.5923); at *K* = 25 for Image 10, MSROA scores 54.8698—nearly 8 units higher than AOA (46.5231) and PDO (46.7252). Moreover, as the threshold *K* increases, the Kapur values of MSROA exhibit a steady upward trend. The growth and performance advantage remain consistent across all thresholds, reflecting MSROA’s adaptability to different parameter settings. In summary, when using Kapur’s entropy as the fitness function, MSROA consistently outperforms most peer algorithms, demonstrating stable and outstanding performance across all evaluation scenarios.

**Table 9:** Statistical results based on Kapur method.

Images	Threshold	MSROA	ROA	SCSO	WOA	PSO	COA	AOA	PDO
1	K=15	**42.5681**	38.1214	41.6667	42.3708	42.3372	42.3830	38.3866	38.2577
	K=20	**50.3164**	44.1861	48.8793	49.9491	49.6340	49.8222	44.2766	44.2194
	K=25	**56.8572**	48.9689	54.8216	56.2494	55.7708	55.8920	49.2027	48.8439
	K=30	**62.4820**	52.7994	59.7445	61.4879	60.9875	60.7553	53.1283	52.8330
2	K=15	**41.9689**	37.5742	41.0128	41.7867	41.6449	41.7807	38.0641	37.5794
	K=20	**49.7342**	43.1018	47.9041	49.4072	49.0339	49.1613	43.5708	43.5265
	K=25	**56.2278**	47.9999	53.6918	55.5748	55.1293	55.2426	48.2633	48.1509
	K=30	**61.8108**	51.9093	58.6175	60.4640	60.6039	60.1738	51.9414	51.9678
3	K=15	**42.5147**	38.2983	41.4859	42.3777	42.3579	42.2672	38.6039	38.3521
	K=20	**50.3869**	44.2670	48.8125	49.9932	49.8262	49.7907	44.5469	44.2315
	K=25	**56.9650**	49.0394	54.9926	56.1227	55.8209	55.9720	49.4565	49.0404
	K=30	**62.6638**	53.3193	59.9618	61.5811	61.3898	60.9375	53.3829	53.2001
4	K=15	**41.9855**	38.1257	41.1634	41.8287	41.7582	41.7680	38.3668	38.0623
	K=20	**49.9763**	44.5059	48.5656	49.6380	49.6308	49.4117	44.4245	44.4232
	K=25	**56.8085**	49.3124	54.9372	56.1416	56.0164	55.7769	49.3652	49.4262
	K=30	**62.6682**	53.4567	60.1010	61.6223	61.3827	60.9556	53.6248	53.7558
5	K=15	**40.7560**	35.7600	39.7563	40.4636	40.1980	40.5628	36.3560	35.5923
	K=20	**47.9727**	40.9659	45.9742	47.5135	46.9709	47.4661	41.3153	40.9598
	K=25	**54.0124**	45.3296	51.3810	53.0869	52.7927	52.9222	45.3640	45.1627
	K=30	**59.0924**	48.8789	55.9792	57.5015	57.3949	57.2074	48.8152	48.5618
6	K=15	**41.4493**	36.4610	40.3425	41.2522	41.0481	41.2683	36.9785	36.4671
	K=20	**49.0239**	42.1889	47.0144	48.5815	48.1563	48.4859	42.4173	42.1809
	K=25	**55.3709**	46.6504	52.6589	54.5131	54.0185	54.2373	46.6612	46.5895
	K=30	**60.7235**	50.5248	57.3995	59.4270	59.1143	58.9184	50.3327	50.5022
7	K=15	**41.6477**	37.1548	40.5457	41.4520	41.3075	41.4101	37.7226	37.1614
	K=20	**49.3635**	42.7964	47.5446	48.7775	48.5496	48.7442	43.1201	42.8026
	K=25	**55.8336**	47.6954	53.5515	54.8572	54.9222	54.7562	47.8268	47.5180
	K=30	**61.4200**	51.5638	58.4087	60.0116	59.9392	59.7187	51.6877	51.4798
8	K=15	**41.4125**	36.8214	40.3990	41.2574	41.0137	41.2399	37.3109	37.0567
	K=20	**49.2256**	43.0931	47.3265	48.7047	48.5110	48.7330	42.7696	42.8788
	K=25	**55.8031**	47.4271	52.8905	55.0546	54.8991	54.8417	47.5825	47.6367
	K=30	**61.3516**	51.5192	57.5548	60.1752	60.0041	59.6834	51.5144	51.4076
9	K=15	**42.9311**	38.7648	41.8752	42.6978	42.7668	42.6122	39.1390	38.8644
	K=20	**51.0277**	45.1028	49.4719	50.6015	50.4498	50.4589	45.2676	45.1398
	K=25	**57.9273**	50.2288	55.8312	57.1202	56.9191	56.8498	50.6536	50.5719
	K=30	**63.7909**	54.8521	60.9884	62.5879	62.9558	62.2460	54.7945	54.8221
10	K=15	**40.7027**	36.2062	39.4686	40.5357	40.4801	40.5208	36.8499	36.2359
	K=20	**48.3677**	42.1226	46.2349	47.9216	47.5494	47.9088	42.1118	42.0755
	K=25	**54.8698**	46.2806	51.7082	54.0938	53.6667	53.9731	46.5231	46.7252
	K=30	**60.1646**	50.4402	56.3567	59.1423	59.0857	58.6541	50.5164	50.3620
11	K=15	**42.8999**	38.7158	42.1059	42.8064	42.6203	42.7433	39.1919	38.8686
	K=20	**50.7173**	44.7365	49.3867	50.4362	50.1183	50.1971	45.1242	44.9100
	K=25	**57.3928**	49.7738	55.2903	56.8007	56.4367	56.3946	49.8925	49.7100
	K=30	**63.1000**	53.5278	60.2148	62.0544	61.6755	61.2918	53.7951	53.7929
12	K=15	**42.3444**	38.2160	41.4092	42.2099	41.9968	42.1475	38.6340	38.0380
	K=20	**50.0735**	43.8066	48.4821	49.6574	49.5535	49.5330	43.9842	43.9366
	K=25	**56.6283**	48.5443	54.4755	55.9861	55.5139	55.5810	48.7937	48.6651
	K=30	**62.1728**	52.6131	59.3595	60.9289	60.8098	60.3791	52.8894	52.6122

#### 5.2.3 Analysis of image segmentation convergence curve.

**Fig 7:**
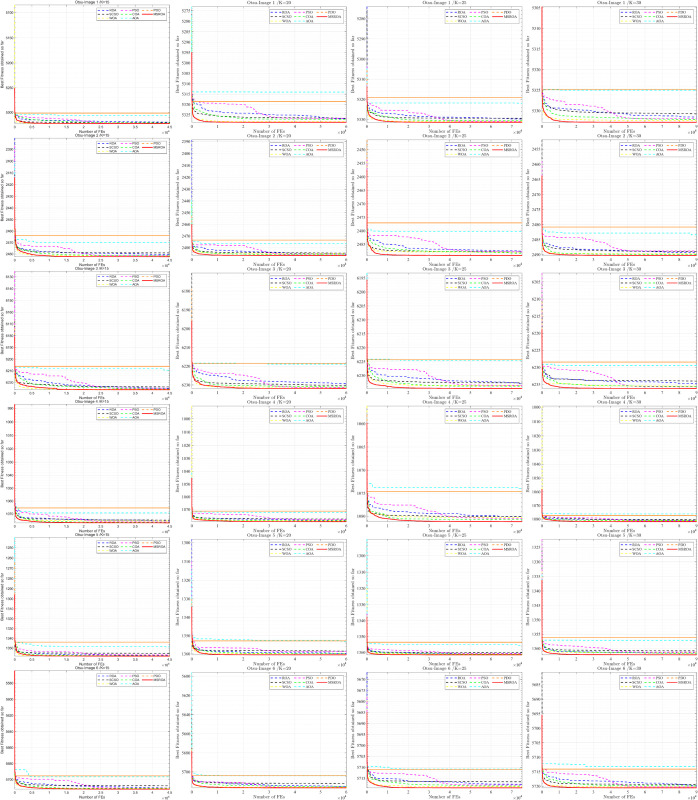
MSROA segmentation results based on Otsu method (Image 1–6.)

**Fig 8:**
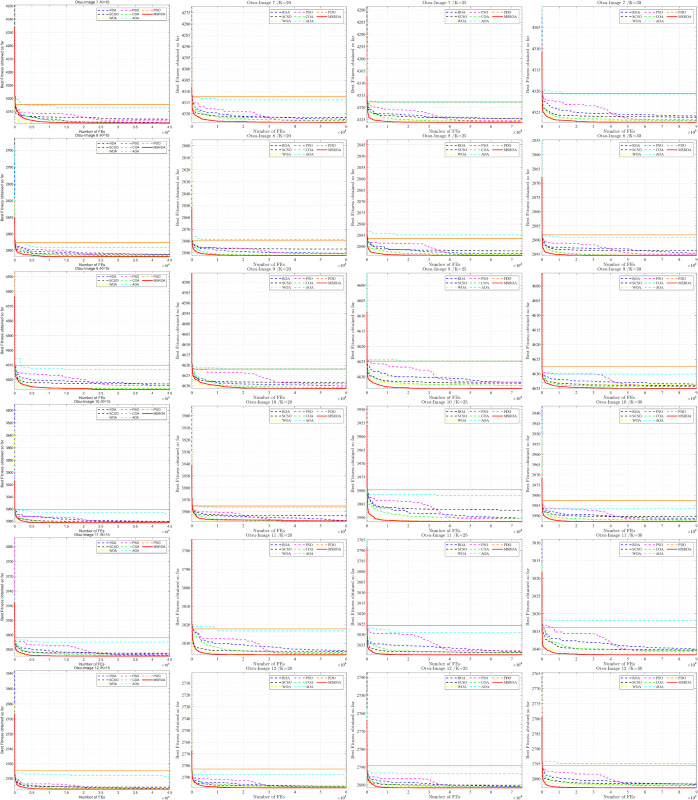
MSROA segmentation results based on Otsu method (Image 7–12).

Figs 7 and 8 present the convergence curves of eight algorithms, including MSROA, ROA, and SCSO, applied to 12 images under four threshold settings (K=15,20,25,30), using Otsu’s entropy as the fitness function. In these curves, higher values indicate better performance. Among all algorithms, MSROA consistently maintains a superior position. Its convergence curves rise rapidly with the number of function evaluations and stabilize at a high level, indicating fast convergence speed and strong final performance. For example, at *K* = 15, the MSROA curve is notably higher than those of the other algorithms across all images. This performance advantage remains evident for *K* = 20, *K* = 25, and *K* = 30, and becomes particularly pronounced in higher-threshold scenarios. In contrast, AOA and PDO show relatively weak performance, with convergence curves remaining at lower levels throughout. The curves of ROA, SCSO, and similar algorithms fall between those of MSROA and the lower-performing methods. As the threshold *K* increases, most algorithms exhibit an upward trend in their convergence curves. However, MSROA demonstrates the most stable and consistent improvement, reflecting its strong adaptability across different segmentation complexities.

**Fig 9:**
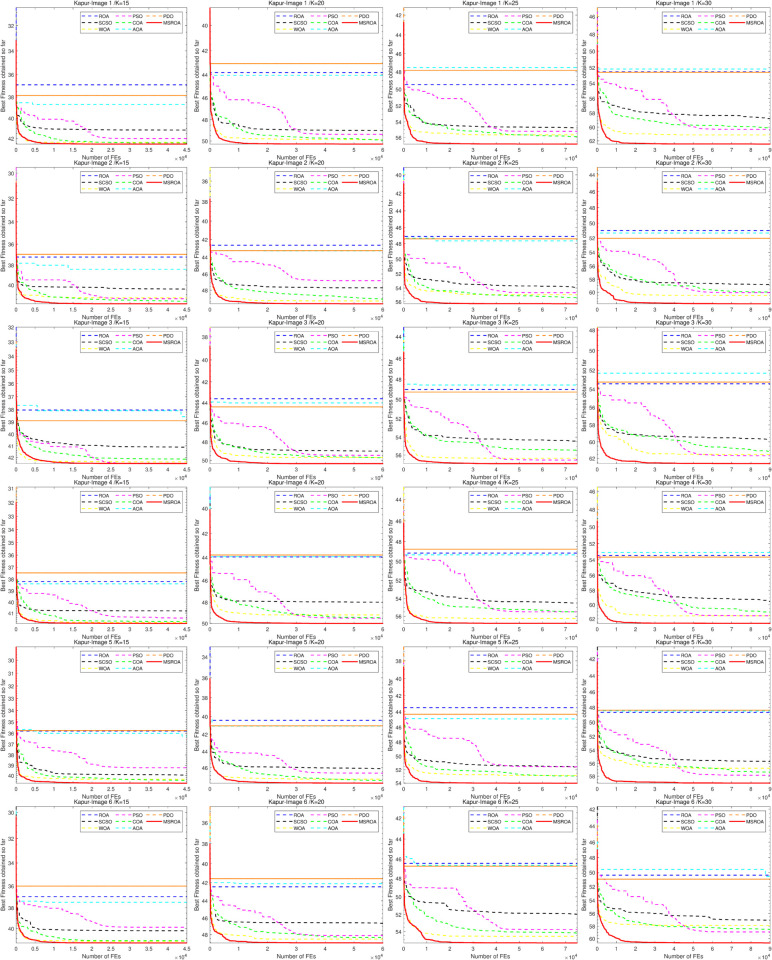
MSROA segmentation results based on kapur method (Image 1–6).

**Fig 10:**
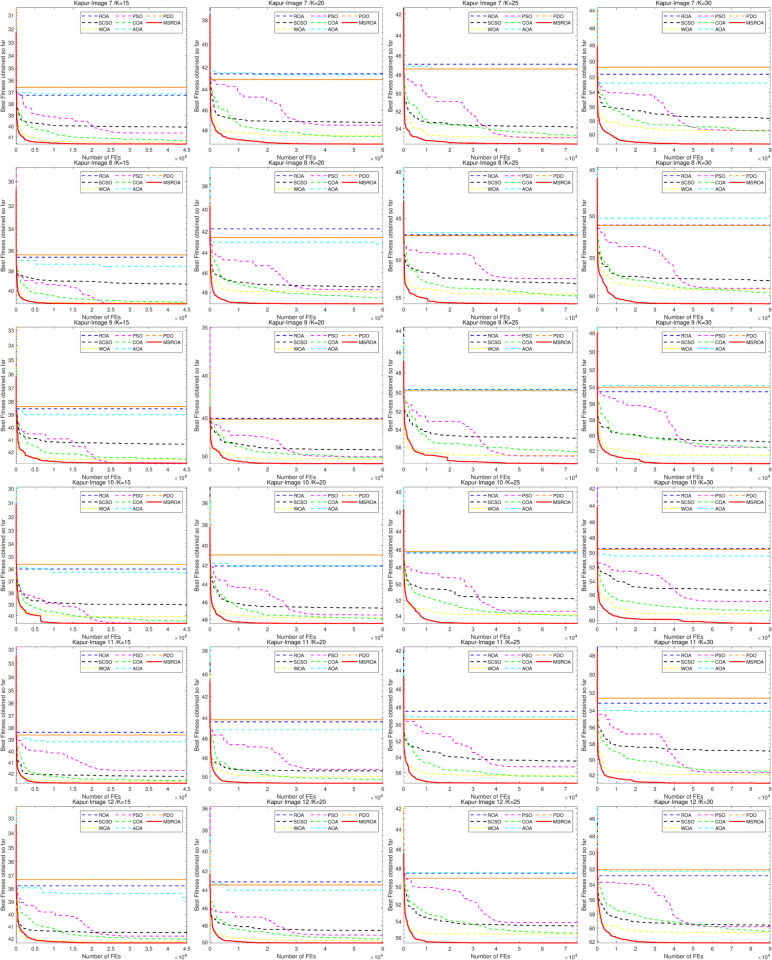
MSROA segmentation results based on kapur method (Image 7–12).

Figs 9 and 10 illustrates the convergence curves of eight algorithms on 12 images under four threshold settings (K=15,20,25,30), using Kapur’s entropy as the fitness function. The higher the curve, the better the algorithm’s performance. Among all methods, MSROA consistently achieves superior performance. Its convergence curves rise rapidly and stabilize early as the number of function evaluations (FEs) increases, demonstrating significant advantages in both convergence speed and final solution quality. When *K* = 15, the MSROA curves are markedly higher than those of other algorithms across all images. This performance advantage persists as the threshold increases to *K* = 20, 25, and 30, particularly under high-threshold settings. In contrast, AOA and PDO generally show weak performance, with convergence curves that remain at lower levels throughout. Algorithms such as ROA and SCSO demonstrate moderate performance, with curve positions and convergence rates lying between those of MSROA and the less competitive methods.

Overall, MSROA outperforms the competing methods in both convergence speed and final optimization results across all scenarios.

### 5.3 Supplementary evaluation indicator

The performance of each algorithm is assessed using a set of quantitative metrics, some of which are employed to evaluate the fitness function during the optimization process, while others are used to assess the quality of the final segmented images. The former is characterized by reporting the arithmetic mean and standard deviation of the fitness values. The latter is evaluated using three widely recognized image quality metrics: the Feature Similarity Index Measure (FSIM), the Peak Signal-to-Noise Ratio (PSNR), and the Structural Similarity Index Measure (SSIM).

FSIM evaluates the perceptual quality of a segmented image by comparing the similarity of structural features between the segmented image and the original image. The specific formulation of FSIM is as follows:

$$FSIM=\frac{\sum_{x\in \Omega }S_{L}(x)\times PC_{m}(x)}{\sum_{x\in \Omega }PC_{m}(x)}$$ (28)

Among them, Ω denotes the entire image pixel domain, *S*_*L*_(*x*) represents the similarity measure of low-level image features at pixel location *x*. *S*_*L*_(*x*) is defined as Eq (29). *PC*_*m*_(*x*) denotes the phase congruency map at *x*, which is defined as $PC_{m}(x)=\max(PC_{1}(x),PC_{2}(x))$. It represents the phase congruency feature derived from the two regions.

$$\begin{array}{c} S_{L}\left(x\right)={\left[S_{PC}\left(x\right)\right]}^{\alpha }\cdot {\left[S_{G}\left(x\right)\right]}^{\beta }\\ S_{{\;\;}_{PC}}\left(x\right)=\frac{2PC_{1}\left(x\right)\times PC_{2}\left(x\right)+T_{1}}{PC_{1}^{2}\left(x\right)\times PC_{2}^{2}\left(x\right)+T_{1}}\\ S_{G}\left(x\right)=\frac{2G_{1}\left(x\right)\times G_{2}\left(x\right)+T_{2}}{G_{1}^{2}\left(x\right)\times G_{2}^{2}\left(x\right)+T_{2}} \end{array}$$ (29)

*S*_*PC*_(*x*) denotes the similarity measure based on phase congruency, while *S*_*G*_(*x*) represents the gradient magnitude similarity between two regions, *G*_1_(*x*) and *G*_2_(*x*). The parameters *α*, *β*, *T*_1_, and *T*_2_ are predefined constants. In this study, to ensure the reproducibility of the experiments and following the standard settings in the literature, the parameters are explicitly set as follows: α=1 and β=1, which assign equal importance to phase congruency and gradient magnitude features. Furthermore, the stability constants are set to *T*_1_ = 0.85 and *T*_2_ = 160.

PSNR is an indicator used to quantify the similarity between the segmented image and the original image. It is defined as:

$$PSNR=10\log_{10}\left(\frac{255^{2}}{MSE}\right)\;MSE=\frac{1}{MN}\sum_{i=1}^{M}\sum_{j=1}^{N}{\left[I(i,j)-K(i,j)\right]}^{2}$$ (30)

Where *MSE* denotes the mean squared error, *I*(*i*, *j*) represents the grayscale value at the *i*th row and *j*th column of the original image, *K*(*i*, *j*) denotes the grayscale value at the corresponding position in the segmented image, and *M* and *N* are the number of rows and columns in the image matrix, respectively.

SSIM evaluates the similarity between two images based on luminance, contrast, and structural information. Its formulation is given by:

$$SSIM(x,y)=\frac{\left(2\mu_{x}\mu_{y}+c_{1}\right)\left(2\sigma_{xy}+c_{2}\right)}{\left(\mu_{x}^{2}+\mu_{y}^{2}+c_{1}\right)\left(\sigma_{x}^{2}+\sigma_{y}^{2}+c_{2}\right)}$$ (31)

Let *x* and *y* denote two images, where $\mu_{x}$ and $\mu_{y}$ represent their respective means, $\sigma_{x}^{2}$ and $\sigma_{y}^{2}$ denote their variances, and $\sigma_{xy}$ is the covariance between *x* and *y*. Constants *c*_1_ and *c*_2_ are included for stability.

#### 5.3.1 Analysis of supplementary evaluation indicators.

Tables 10, 11 and 12 present the performance of the MSROA algorithm using Otsu as the cost function, evaluated in terms of FSIM, PSNR, and SSIM, respectively. Similarly, Tables 13, 14 and 15 report the corresponding results when Kapur entropy is employed as the cost function. Compared with other algorithms such as ROA, WOA, and AOA, MSROA consistently achieves superior numerical performance across all metrics and exhibits a stable, monotonic improvement as the threshold increases. This behavior highlights its stronger adaptability and segmentation efficiency.

**Table 10:** FSIM statistical results based on Otsu method.

Images	Threshold	MSROA	ROA	SCSO	WOA	PSO	COA	AOA	PDO
1	K=15	**0.9692**	0.9636	0.9664	0.9676	0.9655	0.9660	0.9320	0.9230
	K=20	**0.9799**	0.9732	0.9781	0.9778	0.9770	0.9762	0.9490	0.9445
	K=25	**0.9850**	0.9800	0.9839	0.9830	0.9837	0.9816	0.9600	0.9587
	K=30	**0.9882**	0.9828	0.9878	0.9862	0.9868	0.9849	0.9678	0.9662
2	K=15	0.9447	0.9390	**0.9544**	0.9410	0.9352	0.9375	0.9062	0.9036
	K=20	0.9637	0.9526	**0.9719**	0.9612	0.9557	0.9532	0.9265	0.9263
	K=25	0.9756	0.9634	**0.9813**	0.9732	0.9674	0.9670	0.9421	0.9453
	K=30	0.9825	0.9720	**0.9858**	0.9791	0.9761	0.9728	0.9564	0.9576
3	K=15	**0.9293**	0.9197	0.9220	0.9257	0.9248	0.9251	0.8811	0.8741
	K=20	**0.9554**	0.9409	0.9466	0.9495	0.9476	0.9492	0.9030	0.9017
	K=25	**0.9686**	0.9548	0.9594	0.9624	0.9645	0.9615	0.9214	0.9195
	K=30	**0.9772**	0.9633	0.9686	0.9708	0.9729	0.9692	0.9351	0.9348
4	K=15	0.9653	0.9548	**0.9654**	0.9600	0.9565	0.9587	0.9259	0.9157
	K=20	**0.9810**	0.9676	0.9787	0.9731	0.9743	0.9720	0.9424	0.9375
	K=25	**0.9876**	0.9756	0.9845	0.9792	0.9832	0.9798	0.9524	0.9493
	K=30	**0.9915**	0.9809	0.9883	0.9846	0.9881	0.9840	0.9614	0.9580
5	K=15	**0.9803**	0.9744	0.9775	0.9792	0.9751	0.9762	0.9427	0.9254
	K=20	**0.9885**	0.9830	0.9858	0.9865	0.9851	0.9843	0.9566	0.9471
	K=25	**0.9925**	0.9864	0.9893	0.9903	0.9899	0.9886	0.9668	0.9593
	K=30	**0.9945**	0.9890	0.9920	0.9924	0.9925	0.9910	0.9720	0.9687
6	K=15	**0.9339**	0.9235	0.9203	0.9290	0.9232	0.9292	0.8696	0.8520
	K=20	**0.9612**	0.9470	0.9498	0.9554	0.9534	0.9537	0.8948	0.8910
	K=25	**0.9745**	0.9610	0.9664	0.9684	0.9701	0.9666	0.9173	0.9112
	K=30	**0.9822**	0.9692	0.9757	0.9769	0.9784	0.9747	0.9348	0.9295
7	K=15	0.9592	0.9518	**0.9607**	0.9587	0.9546	0.9546	0.9264	0.9217
	K=20	0.9720	0.9641	**0.9750**	0.9684	0.9670	0.9646	0.9398	0.9413
	K=25	0.9785	0.9698	**0.9831**	0.9773	0.9753	0.9709	0.9538	0.9554
	K=30	0.9836	0.9746	**0.9873**	0.9814	0.9791	0.9763	0.9598	0.9620
8	K=15	0.9401	0.9362	**0.9541**	0.9421	0.9352	0.9379	0.9150	0.9179
	K=20	0.9590	0.9519	**0.9714**	0.9587	0.9515	0.9527	0.9340	0.9369
	K=25	0.9722	0.9611	**0.9805**	0.9720	0.9628	0.9644	0.9429	0.9524
	K=30	0.9804	0.9701	**0.9851**	0.9765	0.9710	0.9715	0.9558	0.9610
9	K=15	**0.9523**	0.9435	0.9443	0.9471	0.9457	0.9494	0.9066	0.9019
	K=20	**0.9707**	0.9606	0.9633	0.9648	0.9665	0.9663	0.9313	0.9265
	K=25	**0.9803**	0.9702	0.9739	0.9734	0.9772	0.9752	0.9455	0.9425
	K=30	**0.9862**	0.9758	0.9808	0.9799	0.9836	0.9807	0.9540	0.9524
10	K=15	0.9406	0.9352	**0.9638**	0.9395	0.9320	0.9362	0.9165	0.9239
	K=20	0.9616	0.9569	**0.9791**	0.9617	0.9520	0.9584	0.9441	0.9483
	K=25	0.9746	0.9679	**0.9859**	0.9749	0.9671	0.9721	0.9527	0.9618
	K=30	0.9826	0.9735	**0.9900**	0.9808	0.9764	0.9805	0.9659	0.9715
11	K=15	**0.9644**	0.9590	0.9601	0.9623	0.9603	0.9617	0.9282	0.9169
	K=20	**0.9788**	0.9720	0.9742	0.9760	0.9745	0.9752	0.9458	0.9405
	K=25	**0.9857**	0.9787	0.9826	0.9832	0.9836	0.9821	0.9567	0.9554
	K=30	**0.9896**	0.9837	0.9868	0.9868	0.9882	0.9858	0.9666	0.9635
12	K=15	**0.9816**	0.9781	0.9796	0.9802	0.9784	0.9793	0.9567	0.9435
	K=20	**0.9878**	0.9843	0.9869	0.9862	0.9857	0.9853	0.9660	0.9602
	K=25	**0.9918**	0.9876	0.9907	0.9898	0.9903	0.9885	0.9744	0.9698
	K=30	**0.9936**	0.9901	0.9933	0.9921	0.9925	0.9911	0.9782	0.9769

**Table 11:** PSNR statistical results based on Otsu method.

Images	Threshold	MSROA	ROA	SCSO	WOA	PSO	COA	AOA	PDO
1	K=15	**30.3867**	29.5343	30.1085	29.8233	29.8841	29.6624	27.0105	26.6193
	K=20	**32.4963**	31.1035	32.2323	31.6777	31.7848	31.3857	28.5245	28.2908
	K=25	**34.0602**	32.5542	33.9032	33.0936	33.5355	32.7319	29.9314	29.8193
	K=30	**35.9075**	33.4925	35.3323	34.2321	34.8188	33.8561	30.9128	30.9159
2	K=15	29.7066	28.8338	**30.5091**	28.9323	28.7468	28.6990	26.1810	26.2315
	K=20	32.3173	30.6236	**32.9582**	31.4502	31.2438	30.5918	27.8538	27.9630
	K=25	34.5608	32.1955	**34.8795**	33.3962	33.1081	32.6566	29.4220	29.7399
	K=30	**36.4419**	33.6817	36.2183	34.8115	34.9786	33.8448	31.0791	31.1605
3	K=15	**30.5570**	29.4178	29.5081	29.8383	30.1397	29.7586	26.4045	25.8819
	K=20	**33.0964**	31.4202	32.0455	31.9975	32.3113	31.9977	28.1760	27.9729
	K=25	**35.1424**	33.0492	33.6376	33.6536	34.4449	33.5971	29.7873	29.4927
	K=30	**36.8755**	34.2825	35.0327	34.9961	36.0095	34.8759	31.0199	31.0187
4	K=15	**33.3002**	31.6008	33.2078	32.0867	32.0448	31.9638	28.9435	28.3260
	K=20	**36.6319**	33.6587	35.5914	34.2056	35.0955	34.3209	30.4862	30.3348
	K=25	**38.7544**	35.3099	37.2507	35.8188	37.3901	36.1191	31.5986	31.5561
	K=30	**40.5113**	36.7600	38.6352	37.2498	39.1246	37.6053	32.9996	32.6209
5	K=15	**35.6585**	34.2800	34.7253	34.5519	34.6045	34.3837	30.6533	29.3739
	K=20	**38.2351**	36.2229	36.7042	36.4507	37.0343	36.3909	31.9566	31.1918
	K=25	**40.2986**	37.4640	38.1126	38.0265	38.9465	37.9828	33.1757	32.4994
	K=30	**42.0164**	38.6373	39.5263	39.2403	40.3267	39.2305	34.0083	33.6841
6	K=15	**30.2031**	28.8526	30.1467	29.1728	29.3888	29.1475	26.4149	26.1097
	K=20	**32.6559**	30.7550	32.2796	31.4177	31.7146	31.1926	28.2354	28.1782
	K=25	**34.6017**	32.3931	34.1568	33.0037	33.6736	32.8306	29.5282	29.8117
	K=30	**36.2161**	33.6340	35.5493	34.6277	35.1556	34.3554	31.0542	30.7440
7	K=15	29.5567	28.3173	**29.9585**	29.1684	28.7641	28.5884	26.4932	26.2099
	K=20	32.2358	30.4337	**32.5026**	30.9619	30.9216	30.2738	27.6292	28.1275
	K=25	33.9829	31.7607	**34.6407**	33.0990	33.2528	31.9334	29.4395	29.8471
	K=30	35.6336	33.1636	**36.1545**	34.3779	34.3055	33.3373	30.2930	30.7181
8	K=15	27.2297	26.5537	**28.7152**	27.3373	26.4633	26.5433	24.8008	25.4900
	K=20	30.2087	28.9665	**31.5199**	29.9734	28.9263	28.8501	27.0120	27.7048
	K=25	32.9575	30.6184	**33.5188**	32.5484	30.9180	31.1176	28.2259	29.5489
	K=30	**35.1967**	32.3581	34.9731	33.8055	32.7423	32.7609	30.0308	30.8170
9	K=15	**31.1494**	29.9257	30.0378	30.2525	30.4441	30.4692	27.2566	26.7591
	K=20	**33.7803**	32.0805	32.5304	32.4856	33.1023	32.6761	29.1139	28.8321
	K=25	**35.7796**	33.6074	34.4017	33.9787	35.1332	34.2845	30.5378	30.4626
	K=30	**37.4667**	34.6969	35.9410	35.3665	36.7814	35.5649	31.7518	31.6761
10	K=15	26.4888	25.7115	**28.2224**	26.1488	25.4916	25.7485	24.0954	24.8817
	K=20	29.4248	28.6215	**30.7392**	29.1794	28.0730	28.7425	26.8337	27.1898
	K=25	31.9036	30.4505	**32.4190**	31.6379	30.5019	31.1942	28.0707	29.1429
	K=30	**34.0843**	31.7479	34.0495	33.1550	32.5341	33.0914	29.8999	30.5132
11	K=15	**30.0388**	29.1723	29.4930	29.4127	29.5306	29.3768	26.5427	25.9309
	K=20	**32.5383**	31.1695	31.6011	31.6003	31.7109	31.4508	28.2549	27.8605
	K=25	**34.4769**	32.6424	33.4579	33.2650	33.8250	33.0386	29.5974	29.4974
	K=30	**36.0967**	33.9695	34.8891	34.5426	35.3820	34.2469	30.9098	30.6159
12	K=15	**31.8810**	30.9445	31.2403	31.1053	31.1172	31.0268	28.0852	27.1454
	K=20	**34.1994**	32.8099	33.3196	33.1373	33.3359	32.9162	29.4445	28.9651
	K=25	**36.3089**	34.2090	35.0049	34.7322	35.3615	34.4404	30.7157	30.2528
	K=30	**37.8942**	35.5038	36.4923	36.0347	36.7556	35.6920	31.5621	31.5036

**Table 12:** SSIM statistical results based on Otsu method.

Images	Threshold	MSROA	ROA	SCSO	WOA	PSO	COA	AOA	PDO
1	K=15	0.8630	0.8439	**0.8978**	0.8569	0.8532	0.8410	0.7862	0.7854
	K=20	0.8929	0.8697	**0.9342**	0.8848	0.8870	0.8732	0.8273	0.8276
	K=25	0.9083	0.8982	**0.9501**	0.9036	0.9078	0.8933	0.8701	0.8697
	K=30	0.9445	0.9052	**0.9648**	0.9234	0.9231	0.9073	0.8816	0.8861
2	K=15	0.9178	0.9084	**0.9309**	0.9109	0.9048	0.9059	0.8599	0.8621
	K=20	0.9461	0.9308	**0.9544**	0.9414	0.9353	0.9313	0.8895	0.8936
	K=25	0.9639	0.9469	**0.9676**	0.9586	0.9528	0.9511	0.9138	0.9194
	K=30	**0.9743**	0.9593	0.9740	0.9679	0.9664	0.9607	0.9347	0.9356
3	K=15	**0.9080**	0.8939	0.9052	0.9013	0.9027	0.8982	0.8384	0.8308
	K=20	**0.9418**	0.9237	0.9354	0.9317	0.9322	0.9301	0.8711	0.8702
	K=25	**0.9596**	0.9416	0.9510	0.9491	0.9539	0.9484	0.8984	0.8955
	K=30	**0.9702**	0.9535	0.9615	0.9606	0.9654	0.9587	0.9151	0.9157
4	K=15	0.9442	0.9234	**0.9455**	0.9326	0.9266	0.9296	0.8692	0.8555
	K=20	**0.9732**	0.9487	0.9670	0.9569	0.9615	0.9559	0.9019	0.8949
	K=25	**0.9830**	0.9637	0.9759	0.9688	0.9767	0.9703	0.9193	0.9144
	K=30	**0.9882**	0.9731	0.9820	0.9771	0.9842	0.9781	0.9385	0.9292
5	K=15	**0.9680**	0.9571	0.9641	0.9597	0.9593	0.9558	0.9048	0.8522
	K=20	**0.9818**	0.9716	0.9766	0.9737	0.9760	0.9714	0.9250	0.8976
	K=25	**0.9886**	0.9777	0.9827	0.9809	0.9841	0.9798	0.9391	0.9192
	K=30	**0.9919**	0.9825	0.9872	0.9850	0.9882	0.9843	0.9481	0.9377
6	K=15	0.8668	0.8320	**0.8850**	0.8452	0.8471	0.8413	0.7682	0.7718
	K=20	0.9151	0.8785	**0.9232**	0.8982	0.8974	0.8894	0.8250	0.8302
	K=25	0.9398	0.9106	**0.9455**	0.9233	0.9276	0.9196	0.8539	0.8641
	K=30	0.9549	0.9289	**0.9589**	0.9441	0.9451	0.9415	0.8899	0.8831
7	K=15	0.8903	0.8534	**0.9176**	0.8776	0.8665	0.8604	0.8218	0.8304
	K=20	0.9352	0.9045	**0.9496**	0.9138	0.9109	0.8951	0.8516	0.8722
	K=25	0.9509	0.9251	**0.9658**	0.9455	0.9429	0.9232	0.8909	0.9118
	K=30	0.9620	0.9418	**0.9738**	0.9572	0.9507	0.9408	0.9050	0.9194
8	K=15	0.9197	0.9102	**0.9354**	0.9206	0.9103	0.9133	0.8651	0.8730
	K=20	0.9508	0.9388	**0.9604**	0.9487	0.9405	0.9403	0.9033	0.9064
	K=25	0.9685	0.9534	**0.9723**	0.9663	0.9573	0.9577	0.9198	0.9302
	K=30	0.9776	0.9643	**0.9792**	0.9726	0.9685	0.9675	0.9398	0.9426
9	K=15	**0.9355**	0.9260	0.9318	0.9285	0.9291	0.9311	0.8910	0.8896
	K=20	**0.9592**	0.9465	0.9511	0.9502	0.9546	0.9525	0.9178	0.9162
	K=25	**0.9709**	0.9593	0.9636	0.9611	0.9677	0.9642	0.9334	0.9310
	K=30	**0.9788**	0.9662	0.9719	0.9704	0.9766	0.9716	0.9428	0.9407
10	K=15	0.9274	0.9191	**0.9449**	0.9246	0.9164	0.9204	0.8833	0.8925
	K=20	0.9545	0.9472	**0.9659**	0.9531	0.9438	0.9495	0.9216	0.9263
	K=25	0.9702	0.9606	**0.9757**	0.9689	0.9621	0.9655	0.9363	0.9459
	K=30	0.9794	0.9675	**0.9824**	0.9759	0.9732	0.9755	0.9527	0.9576
11	K=15	0.9332	0.9246	**0.9428**	0.9300	0.9279	0.9259	0.8841	0.8727
	K=20	0.9595	0.9475	**0.9621**	0.9532	0.9518	0.9495	0.9130	0.9077
	K=25	0.9724	0.9593	**0.9739**	0.9673	0.9682	0.9629	0.9305	0.9300
	K=30	**0.9805**	0.9694	0.9804	0.9743	0.9773	0.9706	0.9473	0.9443
12	K=15	0.9446	0.9353	**0.9560**	0.9403	0.9387	0.9354	0.8940	0.8767
	K=20	0.9646	0.9521	**0.9720**	0.9591	0.9570	0.9524	0.9183	0.9117
	K=25	0.9785	0.9644	**0.9811**	0.9699	0.9732	0.9638	0.9337	0.9310
	K=30	0.9835	0.9731	**0.9869**	0.9798	0.9803	0.9738	0.9416	0.9449

**Table 13:** FSIM statistical results based on Kapur entropy method.

Images	Threshold	MSROA	ROA	SCSO	WOA	PSO	COA	AOA	PDO
1	K=15	0.9594	0.9096	**0.9628**	0.9573	0.9590	0.9546	0.9170	0.9079
	K=20	0.9724	0.9319	**0.9753**	0.9709	0.9721	0.9655	0.9372	0.9342
	K=25	**0.9826**	0.9500	0.9817	0.9781	0.9811	0.9724	0.9498	0.9463
	K=30	**0.9887**	0.9596	0.9861	0.9818	0.9863	0.9768	0.9621	0.9583
2	K=15	0.9428	0.8812	**0.9520**	0.9404	0.9425	0.9383	0.8882	0.8838
	K=20	0.9669	0.9150	**0.9677**	0.9604	0.9620	0.9567	0.9220	0.9181
	K=25	**0.9790**	0.9337	0.9768	0.9729	0.9773	0.9691	0.9376	0.9360
	K=30	**0.9849**	0.9503	0.9825	0.9785	0.9841	0.9749	0.9478	0.9494
3	K=15	0.9265	0.8725	0.9235	0.9234	**0.9267**	0.9238	0.8722	0.8698
	K=20	**0.9525**	0.8958	0.9445	0.9471	0.9516	0.9438	0.8999	0.8962
	K=25	**0.9668**	0.9152	0.9582	0.9607	0.9650	0.9576	0.9162	0.9145
	K=30	**0.9760**	0.9299	0.9654	0.9679	0.9737	0.9639	0.9284	0.9278
4	K=15	0.9329	0.8810	0.9335	0.9310	**0.9360**	0.9316	0.8881	0.8821
	K=20	**0.9637**	0.9118	0.9611	0.9567	0.9636	0.9542	0.9081	0.9076
	K=25	**0.9774**	0.9265	0.9730	0.9711	0.9767	0.9652	0.9293	0.9280
	K=30	**0.9847**	0.9405	0.9795	0.9769	0.9839	0.9727	0.9417	0.9389
5	K=15	0.9619	0.8786	**0.9673**	0.9532	0.9610	0.9539	0.8954	0.8880
	K=20	**0.9798**	0.9186	0.9793	0.9726	0.9785	0.9697	0.9145	0.9136
	K=25	**0.9870**	0.9360	0.9849	0.9796	0.9859	0.9757	0.9390	0.9392
	K=30	**0.9909**	0.9496	0.9880	0.9822	0.9903	0.9796	0.9456	0.9461
6	K=15	**0.9133**	0.8348	0.8972	0.9078	0.9104	0.9087	0.8454	0.8415
	K=20	**0.9515**	0.8648	0.9359	0.9447	0.9476	0.9416	0.8756	0.8667
	K=25	**0.9705**	0.8943	0.9570	0.9621	0.9669	0.9566	0.8879	0.8942
	K=30	**0.9805**	0.9145	0.9705	0.9714	0.9779	0.9661	0.9163	0.9186
7	K=15	0.9439	0.9010	**0.9562**	0.9389	0.9426	0.9367	0.9053	0.8990
	K=20	0.9673	0.9251	**0.9717**	0.9594	0.9643	0.9548	0.9278	0.9273
	K=25	**0.9803**	0.9450	0.9774	0.9698	0.9773	0.9680	0.9446	0.9439
	K=30	**0.9865**	0.9569	0.9840	0.9773	0.9854	0.9768	0.9539	0.9541
8	K=15	0.9556	0.9013	**0.9591**	0.9520	0.9532	0.9510	0.9082	0.9035
	K=20	**0.9752**	0.9335	0.9727	0.9701	0.9743	0.9685	0.9344	0.9319
	K=25	**0.9832**	0.9441	0.9792	0.9790	0.9830	0.9762	0.9441	0.9465
	K=30	**0.9882**	0.9540	0.9829	0.9837	0.9876	0.9808	0.9571	0.9575
9	K=15	**0.9415**	0.8869	0.9362	0.9342	0.9408	0.9367	0.8933	0.8840
	K=20	**0.9621**	0.9102	0.9544	0.9565	0.9609	0.9536	0.9118	0.9153
	K=25	**0.9737**	0.9293	0.9657	0.9675	0.9724	0.9670	0.9322	0.9331
	K=30	**0.9808**	0.9421	0.9735	0.9736	0.9799	0.9728	0.9433	0.9432
10	K=15	**0.9763**	0.9247	0.9672	0.9749	0.9759	0.9712	0.9305	0.9218
	K=20	**0.9861**	0.9481	0.9806	0.9837	0.9853	0.9810	0.9485	0.9483
	K=25	**0.9908**	0.9589	0.9855	0.9878	0.9901	0.9849	0.9604	0.9582
	K=30	**0.9933**	0.9680	0.9882	0.9897	0.9927	0.9883	0.9691	0.9679
11	K=15	**0.9640**	0.9077	0.9617	0.9620	0.9632	0.9598	0.9117	0.9104
	K=20	**0.9785**	0.9311	0.9757	0.9753	0.9777	0.9728	0.9361	0.9334
	K=25	**0.9871**	0.9505	0.9825	0.9825	0.9861	0.9788	0.9501	0.9503
	K=30	**0.9911**	0.9585	0.9865	0.9859	0.9903	0.9821	0.9603	0.9601
12	K=15	0.9741	0.9230	**0.9778**	0.9711	0.9734	0.9685	0.9294	0.9183
	K=20	0.9857	0.9474	**0.9862**	0.9814	0.9850	0.9784	0.9477	0.9425
	K=25	**0.9913**	0.9589	0.9900	0.9864	0.9905	0.9828	0.9606	0.9587
	K=30	**0.9937**	0.9700	0.9926	0.9891	0.9934	0.9855	0.9694	0.9694

**Table 14:** PSNR statistical results based on Kapur entropy method.

Images	Threshold	MSROA	ROA	SCSO	WOA	PSO	COA	AOA	PDO
1	K=15	28.7987	25.7384	**29.5613**	28.4395	28.7632	28.1438	26.1694	25.6329
	K=20	30.7771	27.4848	**31.7412**	30.3713	30.8512	29.7463	27.7154	27.5555
	K=25	33.3621	29.0508	**33.4132**	32.0410	32.8768	31.1521	29.0718	28.8068
	K=30	**35.3845**	30.2430	34.8794	33.1474	34.5549	32.2319	30.5230	30.1726
2	K=15	28.7267	24.6055	**29.3504**	28.1798	28.6755	28.0881	25.0644	24.7582
	K=20	**31.7521**	27.0427	31.5997	30.5888	31.1837	30.3253	27.3421	27.1486
	K=25	**34.0009**	28.6564	33.3445	32.5972	33.7063	32.1864	28.9085	28.6309
	K=30	**35.6062**	30.2812	34.9008	33.7733	35.4071	33.4281	30.0145	30.1492
3	K=15	29.4060	25.4782	28.2642	28.9054	**29.4825**	28.7796	25.6408	25.4275
	K=20	**31.9550**	27.2414	30.3621	31.1119	31.7978	30.7180	27.3619	27.1980
	K=25	**34.0196**	28.8428	31.9731	32.8068	33.7728	32.5414	28.8271	28.7211
	K=30	**35.7786**	30.1013	33.2023	34.0181	35.4109	33.4625	30.0071	29.9690
4	K=15	28.9710	25.9064	28.8397	28.7397	**29.2889**	28.7921	26.3899	25.8781
	K=20	**31.9022**	28.0711	31.5940	30.8553	31.8576	30.8770	27.7859	27.6249
	K=25	**33.9722**	29.3812	33.2963	32.6940	33.8108	32.2540	29.3941	29.3676
	K=30	**35.5349**	30.5979	34.7771	33.8909	35.3106	33.5518	30.7477	30.2371
5	K=15	30.8342	26.9186	**31.9822**	30.0028	30.7717	30.1346	27.7147	27.2211
	K=20	33.4916	29.0310	**34.1220**	32.3313	33.2448	32.1223	28.5866	28.7640
	K=25	35.4184	30.5925	**35.6524**	33.7342	35.0839	33.3867	30.5014	30.3163
	K=30	37.0158	31.3743	**37.0316**	34.9283	36.7368	34.4411	31.1036	31.0773
6	K=15	**29.5538**	25.3294	29.2839	28.7974	29.2982	28.6143	25.7311	25.4799
	K=20	**32.4455**	27.2008	31.6629	31.1639	32.0096	30.9098	27.5399	27.2049
	K=25	**34.4596**	28.7401	33.4206	32.9595	33.9501	32.2749	28.5801	28.9200
	K=30	**36.2249**	30.1404	34.9097	34.2253	35.6652	33.6164	30.2593	30.3315
7	K=15	27.8283	25.1744	**28.9242**	27.3474	27.7412	27.0520	25.1857	25.0630
	K=20	31.0947	26.7782	**31.3121**	30.0779	30.6764	29.2300	27.1045	27.0934
	K=25	**33.7672**	28.7401	32.9794	31.7875	33.0322	31.5891	28.6421	28.6097
	K=30	**35.5571**	30.1239	34.6343	33.3979	35.2659	33.2584	29.9689	30.0549
8	K=15	**29.3313**	24.5944	29.1314	28.5818	29.0446	28.4458	25.0684	24.7800
	K=20	**32.0886**	27.3172	31.2994	31.0779	31.8868	30.9994	27.4330	27.2007
	K=25	**34.1588**	28.6261	32.7591	32.9365	34.0382	32.5910	28.5062	28.6649
	K=30	**35.8183**	29.7560	33.8194	34.1791	35.5211	33.9189	30.0369	30.2352
9	K=15	**28.8358**	25.1038	27.6581	28.3255	28.5902	28.4465	25.3335	25.1316
	K=20	31.7379	26.9565	29.9384	31.0044	**31.7595**	30.8255	27.2594	26.9718
	K=25	**33.8298**	28.6282	31.8782	32.5725	33.5182	32.6005	28.7815	29.0336
	K=30	**35.5153**	29.8540	33.5173	33.8955	35.2440	33.7133	30.2505	29.8183
10	K=15	**29.7336**	25.0322	27.3743	29.2057	29.6574	28.7784	25.2412	24.8215
	K=20	**32.3988**	27.1840	29.8901	31.5198	32.1462	31.1346	27.1497	27.0827
	K=25	**34.5588**	28.5517	31.5218	33.3453	34.2006	32.6557	28.8202	28.6314
	K=30	**36.1449**	29.9198	32.6641	34.5417	35.8577	33.9492	29.9971	29.8588
11	K=15	**29.6507**	25.2078	29.4487	29.0613	29.5649	28.9229	25.4891	25.3631
	K=20	**31.9938**	27.1172	31.6894	31.1143	31.8578	30.7960	27.4652	27.2421
	K=25	**34.1395**	28.9344	33.2723	32.7311	33.8804	32.2106	28.8843	28.9482
	K=30	**35.7423**	29.9621	34.6289	33.9296	35.4095	33.2193	30.0637	30.2409
12	K=15	29.7094	25.8569	**30.2557**	29.0995	29.5970	28.9742	26.2084	25.5642
	K=20	32.3785	27.7931	**32.5161**	31.3587	32.2393	30.9455	27.8159	27.3972
	K=25	**34.4675**	29.0856	34.0982	32.9109	34.1651	32.3594	29.2310	28.9210
	K=30	**36.1316**	30.6060	35.6893	34.1588	35.8260	33.3633	30.4182	30.4507

**Table 15:** SSIM statistical results based on Kapur entropy method.

Images	Threshold	MSROA	ROA	SCSO	WOA	PSO	COA	AOA	PDO
1	K=15	0.7682	0.7425	**0.8603**	0.7863	0.7708	0.7536	0.7566	0.7356
	K=20	0.8149	0.8105	**0.9128**	0.8347	0.8344	0.7955	0.8046	0.7952
	K=25	0.9136	0.8338	**0.9387**	0.8823	0.8936	0.8438	0.8322	0.8358
	K=30	0.9500	0.8743	**0.9583**	0.9010	0.9284	0.8701	0.8844	0.8722
2	K=15	0.9077	0.8370	**0.9217**	0.9034	0.9068	0.9003	0.8477	0.8435
	K=20	0.9404	0.8823	**0.9416**	0.9329	0.9351	0.9284	0.8904	0.8855
	K=25	**0.9584**	0.9069	0.9551	0.9509	0.9562	0.9468	0.9104	0.9088
	K=30	**0.9682**	0.9259	0.9648	0.9601	0.9668	0.9569	0.9235	0.9255
3	K=15	0.8925	0.8307	**0.8995**	0.8877	0.8933	0.8903	0.8300	0.8282
	K=20	**0.9295**	0.8623	0.9245	0.9217	0.9290	0.9180	0.8664	0.8637
	K=25	**0.9500**	0.8906	0.9445	0.9411	0.9481	0.9395	0.8879	0.8852
	K=30	**0.9622**	0.9068	0.9543	0.9514	0.9599	0.9463	0.9060	0.9037
4	K=15	0.8707	0.8042	0.8687	0.8696	**0.8750**	0.8710	0.8125	0.8043
	K=20	**0.9166**	0.8501	0.9117	0.9077	0.9159	0.9071	0.8405	0.8422
	K=25	**0.9434**	0.8741	0.9338	0.9340	0.9414	0.9266	0.8765	0.8748
	K=30	**0.9582**	0.8962	0.9484	0.9471	0.9564	0.9424	0.8970	0.8912
5	K=15	0.8512	0.7242	**0.9135**	0.8383	0.8470	0.8210	0.7551	0.7599
	K=20	0.9193	0.8126	**0.9448**	0.8985	0.9100	0.8747	0.7887	0.7905
	K=25	0.9485	0.8538	**0.9588**	0.9268	0.9398	0.9034	0.8440	0.8449
	K=30	0.9637	0.8703	**0.9684**	0.9355	0.9612	0.9205	0.8581	0.8609
6	K=15	0.8495	0.7615	**0.8682**	0.8414	0.8403	0.8286	0.7709	0.7627
	K=20	**0.9148**	0.8183	0.9147	0.8931	0.9046	0.8849	0.8168	0.8152
	K=25	**0.9405**	0.8515	0.9384	0.9237	0.9337	0.9104	0.8493	0.8541
	K=30	**0.9575**	0.8793	0.9534	0.9396	0.9526	0.9306	0.8807	0.8822
7	K=15	0.8717	0.8038	**0.9021**	0.8578	0.8711	0.8366	0.8077	0.8120
	K=20	0.9243	0.8605	**0.9397**	0.9031	0.9165	0.8827	0.8522	0.8567
	K=25	**0.9567**	0.8959	0.9545	0.9325	0.9460	0.9295	0.8907	0.8910
	K=30	**0.9683**	0.9132	0.9657	0.9509	0.9666	0.9449	0.9104	0.9161
8	K=15	0.9203	0.8403	**0.9221**	0.9157	0.9167	0.9154	0.8476	0.8412
	K=20	**0.9518**	0.8878	0.9495	0.9460	0.9498	0.9457	0.8926	0.8873
	K=25	**0.9669**	0.9069	0.9636	0.9611	0.9666	0.9593	0.9048	0.9097
	K=30	**0.9761**	0.9199	0.9707	0.9697	0.9749	0.9681	0.9261	0.9291
9	K=15	0.9288	0.8774	**0.9304**	0.9188	0.9278	0.9215	0.8841	0.8738
	K=20	**0.9464**	0.8974	0.9438	0.9383	0.9448	0.9347	0.8986	0.9054
	K=25	**0.9572**	0.9159	0.9534	0.9500	0.9560	0.9496	0.9191	0.9186
	K=30	**0.9662**	0.9270	0.9616	0.9572	0.9650	0.9568	0.9294	0.9312
10	K=15	**0.9573**	0.8942	0.9454	0.9552	0.9568	0.9527	0.9016	0.8916
	K=20	**0.9735**	0.9247	0.9643	0.9702	0.9721	0.9680	0.9256	0.9250
	K=25	**0.9821**	0.9393	0.9734	0.9782	0.9809	0.9753	0.9407	0.9396
	K=30	**0.9869**	0.9513	0.9789	0.9822	0.9859	0.9804	0.9530	0.9515
11	K=15	0.9292	0.8611	**0.9428**	0.9256	0.9285	0.9198	0.8649	0.8660
	K=20	0.9573	0.8928	**0.9633**	0.9512	0.9558	0.9458	0.9008	0.9006
	K=25	**0.9755**	0.9239	0.9734	0.9649	0.9733	0.9574	0.9243	0.9249
	K=30	**0.9820**	0.9381	0.9793	0.9719	0.9807	0.9645	0.9386	0.9418
12	K=15	0.9028	0.8430	**0.9441**	0.9010	0.9038	0.8885	0.8417	0.8327
	K=20	0.9510	0.8843	**0.9657**	0.9383	0.9481	0.9245	0.8837	0.8768
	K=25	0.9736	0.9094	**0.9762**	0.9557	0.9698	0.9403	0.9112	0.9087
	K=30	0.9807	0.9312	**0.9830**	0.9659	0.9797	0.9512	0.9307	0.9237

Across all metrics—FSIM (reflecting phase congruency), PSNR (quantifying grayscale fidelity), and SSIM (assessing structural similarity)—MSROA demonstrates robust segmentation performance under different fitness functions. Its overall effectiveness significantly exceeds that of the comparative algorithms.

While MSROA outperforms in most scenarios, some competing algorithms also demonstrate competitive performance under specific conditions. For instance, the SCSO algorithm yields results close to MSROA in certain SSIM evaluations using the Otsu method. In Image 2, at *K* = 30, SCSO achieves an SSIM value of 0.9743, only 0.0003 below the 0.9780 obtained by MSROA. However, SCSO’s performance across all images and thresholds lacks the stability observed in MSROA.

Similarly, the PSO algorithm achieves competitive results in specific FSIM evaluations under the Kapur entropy criterion. For example, for Image 10 at *K* = 15, PSO attains an FSIM of 0.9360, slightly higher than MSROA’s 0.9329. Nevertheless, PSO’s performance is less consistent across different thresholds and metrics.

In summary, due to its stable and high numerical performance across diverse cost functions and evaluation criteria, MSROA exhibits a significant advantage in image segmentation and consistently outperforms most other algorithms in comprehensive evaluations.

## 6 Conclusion

ROA, a meta-heuristic method with notable search performance, suffers from drawbacks such as slow convergence and limited accuracy due to its reliance on random host selection. To address these limitations, this study introduces a MSROA. MSROA integrates a Beta random restart strategy with prior-guided properties, a random walk and fast predation mechanism, and an elite learning strategy to enhance both convergence speed and solution accuracy. To comprehensively evaluate its performance, MSROA is first benchmarked against standard test suites from CEC2017 and CEC2020. Comparative analysis with seven well-established algorithms confirms the strong optimization capabilities of MSROA. In real-world applications, MSROA is applied to multi-threshold image segmentation tasks. By using Otsu’s method and Kapur’s entropy as objective functions, it effectively identifies optimal threshold combinations for color image segmentation. In this context, MSROA not only achieves the highest values for the chosen objective functions but also delivers superior segmentation results. Quantitative evaluations demonstrate that when assessed using Peak Signal-to-Noise Ratio (PSNR), Feature Similarity Index Measure (FSIM), and Structural Similarity Index Measure (SSIM), MSROA consistently achieves the highest average scores compared to other algorithms for both Otsu and Kapur methods. The data confirms that MSROA yields segmentation results with higher structural fidelity and lower distortion, particularly at higher threshold levels.

Future work will focus on further enhancing MSROA by incorporating concepts such as chaos theory, stochastic processes, game theory, and biological population competition models. These improvements aim to develop a more efficient and general-purpose optimization algorithm. Additionally, MSROA will be extended to more complex image processing tasks and broader optimization domains, offering a robust solution for a wide range of practical problems.
